# A negative feedback loop controls NMDA receptor function in cortical interneurons via neuregulin 2/ErbB4 signalling

**DOI:** 10.1038/ncomms8222

**Published:** 2015-06-01

**Authors:** Detlef Vullhorst, Robert M. Mitchell, Carolyn Keating, Swagata Roychowdhury, Irina Karavanova, Jung-Hwa Tao-Cheng, Andres Buonanno

**Affiliations:** 1 Section on Molecular Neurobiology, Eunice Shriver Kennedy National Institute of Child Health and Human Development, Bethesda, Maryland 20892, USA; 2 EM Facility, National Institute of Neurological Disorders and Stroke, Bethesda, Maryland 20892, USA

## Abstract

The neuregulin receptor ErbB4 is an important modulator of GABAergic interneurons and neural network synchronization. However, little is known about the endogenous ligands that engage ErbB4, the neural processes that activate them or their direct downstream targets. Here we demonstrate, in cultured neurons and in acute slices, that the NMDA receptor is both effector and target of neuregulin 2 (NRG2)/ErbB4 signalling in cortical interneurons. Interneurons co-express ErbB4 and NRG2, and pro-NRG2 accumulates on cell bodies atop subsurface cisternae. NMDA receptor activation rapidly triggers shedding of the signalling-competent NRG2 extracellular domain. In turn, NRG2 promotes ErbB4 association with GluN2B-containing NMDA receptors, followed by rapid internalization of surface receptors and potent downregulation of NMDA but not AMPA receptor currents. These effects occur selectively in ErbB4-positive interneurons and not in ErbB4-negative pyramidal neurons. Our findings reveal an intimate reciprocal relationship between ErbB4 and NMDA receptors with possible implications for the modulation of cortical microcircuits associated with cognitive deficits in psychiatric disorders.

## Abstract (web summary)

The neuregulin receptor ErbB4 is an important modulator of GABAergic interneurons and neural network synchronization but little is known about the endogenous ligands that engage ErbB4 and the downstream targets. Here the authors describe the existence of a cell-autonomous bidirectional pathway that links NMDA receptor activity with NRG signalling in GABAergic interneurons.

## Graphical abstract

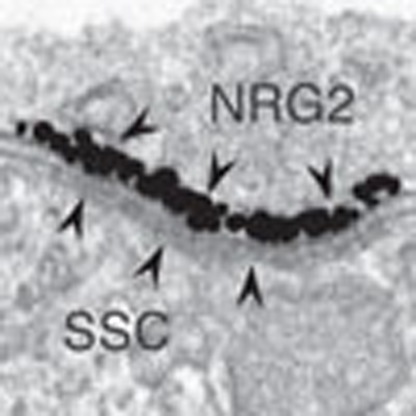

Local GABA(gamma aminobutyric acid)-ergic inhibitory interneurons are essential coordinators of cortical microcircuits and are implicated in epilepsy, schizophrenia and other pathologies in which the balance of excitatory and inhibitory transmission is perturbed. Because of their critical role in modulating neuronal network activity by transiently entraining groups of principal neurons into synchronously firing ensembles, it is important to understand how this extremely diverse class of neurons is itself regulated by interactions between fast-acting synaptic transmission and slow-acting neuromodulators. Two such regulators that have received much attention are *N*-methyl-D-aspartate receptors (NMDARs) and the receptor tyrosine kinase ErbB4. NMDARs expressed on interneurons figure prominently in the glutamate hypofunction theory of schizophrenia^1^,^2^,^3^, and were shown by interneuron-restricted gene ablation in mice to be essential for normal local network synchrony and behaviour^4^,^5^,^6^.

The ErbB4 receptor is widely expressed in GABAergic interneurons but is undetectable in pyramidal neurons^7^,^8^. Stimulation of ErbB4 in acute slice preparations by recombinant neuregulin 1 (NRG1) encompassing the minimal epidermal-like growth factor(EGF)-like domain affects GABAergic transmission onto pyramidal neurons, gamma oscillations and pyramidal neuron synaptic plasticity^9^,^10^,^11^,^12^,^13^. Studies on the effects of NRGs directly on ErbB4-positive (ErbB4^+^) interneurons indicate that acute stimulation of ErbB4 promotes the internalization of ion channels from the cell surface^14^,^15^,^16^. Although prior studies identified interactions between ErbB4 and PSD95 at glutamatergic postsynaptic densities^9^,^17^, an involvement of acute NRG/ErbB4 signalling in the regulation of glutamatergic transmission onto inhibitory interneurons has not been reported.

While significant progress has been made in delineating the effects of ErbB4 signalling on cortical circuits^18^,^19^, far less is known about the functionally relevant endogenous NRG ligands that engage ErbB4 during neural processes in the adolescent and adult central nervous system (CNS). For historical reasons, NRG1-derived recombinant protein fragments have most commonly been used to study ErbB4 functions, although postnatal NRG1 mRNA expression in the CNS is generally lower than the closely related NRG2 isoform^20^, consistent with the notion that NRG2 is a major functional ErbB4 ligand in the postnatal brain. Using the EGF-like domain of NRG is convenient but bypasses important aspects of its biology. Most NRGs are synthesized as single-pass transmembrane pro-forms that must be converted to signalling-competent receptor ligands by extracellular sheddases. The presence of an immunoglobulin (Ig)-like domain in most NRG1 isoforms and in NRG2 allows the ectodomain to accumulate near the site of cleavage through interactions with extracellular matrix heparan proteoglycans^21^. By contrast, cysteine-rich-domain(CRD)-NRG1 has a second transmembrane domain upstream of the EGF-like domain and is thought to signal as a transmembrane ligand^22^. While critical for our understanding of NRG/ErbB4 signalling, it is unclear whether and how NRG ectodomain shedding is regulated in the CNS.

Several studies have linked the NRG/ErbB4 pathway and NMDAR function in pyramidal neurons (see, for example, ref. 23). However, given that ErbB4 receptors are undetectable in hippocampal and neocortical pyramidal neurons^7^,^8^, and that interneuron NMDARs play an important role in cortical network synchrony^4^,^5^,^6^, and in the glutamate hypothesis of schizophrenia^1^,^2^,^3^ a better understanding of the direct effects of NRG/ErbB4 signalling on interneuron NMDARs is warranted. In this work, we describe the existence of a cell-autonomous pathway that intimately and bidirectionally links NMDAR activity with NRG signalling in GABAergic interneurons. We demonstrate that NMDARs are required to release the biologically active ectodomain of NRG2 from clustered aggregates on the surface of interneuron cell bodies. In turn, stimulation of ErbB4 by the NRG2 ectodomain promotes the association of ErbB4 with GluN2B-containing NMDARs, and rapidly and potently downregulates NMDARs on inhibitory interneurons but not pyramidal neurons. Taken together, our findings provide the foundation for a new understanding of the functional relationship between these important receptor systems and their roles in the modulation of interneurons and local circuit functions.

## Results

### NRG2 is expressed in ErbB4^+^ interneurons

**Figure 1:**
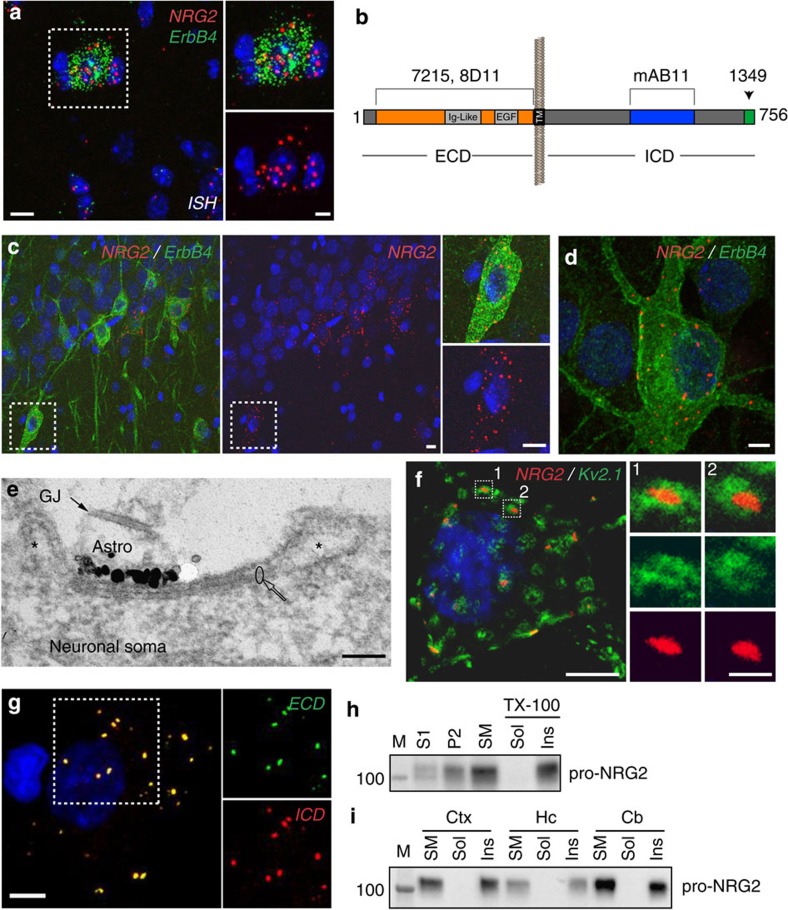
Pro-NRG2 protein accumulates atop subsurface cisternae in ErbB4-positive interneurons. (**a**) Double-label fluorescence ISH of NRG2 and ErbB4 in the mouse hippocampus, showing overlapping signals in a neuron located in the stratum oriens of area CA1. The pyramidal cell layer is visible in the lower right corner. DAPI was added to label nuclei (blue). Note that ErbB4-negative cells have much lower or no NRG2 signal. Magnified areas on the right outlined by boundary box. (**b**) Immunogen sites in NRG2 used to raise poly- and monoclonal NRG2 antibodies. Ig-like and EGF-like domains in the ECD, the transmembrane (TM) and ICD are shown. (**c**) NRG2 immunoreactivity in ErbB4^+^ interneurons in rat CA1 strata pyramidale and radiatum. (**d**) Somatodendritic NRG2 surface puncta on a DIV 28 cultured ErbB4^+^ hippocampal neuron. (**e**) Immunogold electron micrograph of a DIV 35 hippocampal neuron with a patch of concentrated label for NRG2 (black particles depict silver-enhanced signals) at the plasma membrane atop intracellular membrane stacks characteristic of SSCs. Asterisks mark the lumen of the open cisterns that are continuous and surround the flattened stacks of the specialized endoplasmic reticulum (ER) membranes (circle) in the centre of the SSC. Astroglial processes (astro, identified by a gap junction (GJ) between two processes) are often closely associated with SSCs. (**f**) Super-resolution microscopy image of a 2-month old hippocampal neuron double-labelled for NRG2 and Kv2.1. Magnified areas illustrate how NRG2 frequently resides in the centre of doughnut-shaped Kv2.1 clusters. (**g**) Immunofluorescence signals obtained with ECD antibody 8D11 (surface-labelled) and ICD antibody mAB11 exhibit extensive overlap, suggesting that puncta consist of unprocessed pro-NRG2. (**h**,**i**) The ∼110 kDa pro-NRG2 protein is enriched in SMs and partitions with the Triton X-100 (TX-100) insoluble fraction in the rat cortex (Ctx), hippocampus (Hc) and cerebellum (Cb). NRG2 was detected with polyclonal antibody 1349 against the carboxyl terminus. M, molecular mass standards; S1, crude extract; P2, crude membranes; SM, synaptosomal membranes; Sol, detergent-soluble; Ins, detergent-insoluble. Scale bars, 10 μm (**a**,**c**), 5 μm (**d**,**f**,**g** and inset in **a**), 1 μm (inset in **f**), 0.1 μm (**e**). Image shown in **a** is representative of two experiments, micrographs in **c**–**g** are representative of at least three replicates.

Regional NRG2 expression patterns in the rodent CNS have largely been investigated at the mRNA level^20^,^24^, with highest transcript levels in granule cells of the cerebellum, hippocampus and olfactory bulb. To better understand the cellular expression pattern of NRG2 mRNA in relationship to its cognate receptor ErbB4, we used double-label fluorescence *in situ* hybridization (ISH) of the mouse hippocampus with probes for NRG2 and ErbB4. While weak NRG2 signals were detected in many cells, much higher levels were consistently detected in ErbB4^+^ cells (Fig. 1a), indicating that ErbB4^+^ interneurons co-express NRG2. These findings were corroborated using a probe for Gad1 (Supplementary Fig. 1). Intrigued by the possibility of an autocrine NRG2/ErbB4 signalling loop in GABAergic interneurons, we developed mono- and polyclonal antibodies against the extra (ECD)- and intracellular (ICD) domains of NRG2 (Fig. 1b and Supplementary Fig. 2). Both mouse and rabbit monoclonal antibodies raised against the NRG2-ECD and ICD, respectively, are specific for NRG2 and do not crossreact with NRG1 (Supplementary Figs 3 and 4). Using mouse monoclonal antibody 8D11 against the ECD, we indeed found prominent NRG2 signals in the soma and proximal dendrites of ErbB4^+^ interneurons in hippocampal areas CA1–CA3 (Fig. 1c). A representative count of NRG2^+^ cells (excluding dentate gyrus granule cells) from three rats revealed that of 558 NRG2^+^ neurons, 454 versus 104 (81.4%) co-expressed ErbB4. Neurons positive for ErbB4 and negative for NRG2 were virtually absent.

### Pro-NRG2 protein accumulates atop subsurface cisternae

We also observed somatodendritic NRG2 puncta on cultured ErbB4^+^ interneurons using antibody 8D11 under non-permeabilizing conditions, indicating that these puncta reside at the cell surface (Fig. 1d). We further investigated this subcellular distribution by first testing for potential co-localization of NRG2 puncta with the presynaptic marker bassoon, the postsynaptic excitatory synapse marker PSD95 and the postsynaptic inhibitory synapse marker gephyrin, but found no overlap (Supplementary Fig. 5). Exploring other possibilities, we noticed that NRG2 puncta were reminiscent of the distribution of subsurface cisternae (SSC), flattened extensions of the endoplasmic reticulum located immediately adjacent to the plasma membrane^25^. Based on data indicating that NRG2 puncta number and size increase with culture age (Supplementary Fig. 6), we used silver-enhanced immunogold electron microscopy in aged (35 days *in vitro*; DIV 35) hippocampal neurons. This approach revealed highly concentrated patches of NRG2 at the outer surface of neuronal soma plasma membrane atop intracellular SSCs, which are typically apposed by astrocytic processes (Fig. 1e), or sometimes by axon terminals (Supplementary Fig. 7).

We corroborated these findings by double immunofluorescence of NRG2 and Kv2.1, a voltage-gated potassium channel that forms large clusters near SSCs on the cell body and proximal dendrites of numerous neuron types including cultured GABAergic interneurons^26^,^27^. Stimulated emission depletion (STED) and structured illumination (SIM) super-resolution microscopy of hippocampal neurons revealed a close spatial relationship between the micron-size Kv2.1 clusters and the more compact NRG2 puncta, the latter frequently residing in the open centres of Kv2.1 clusters (‘doughnut’ holes; Fig. 1f and Supplementary Movie 1 for a three-dimensional (3D) reconstruction). These observations correlate well with the electron microscpoy (EM) findings that label for Kv2.1 is concentrated at the open cisterns at the periphery of the disc-like SSC^26^, while label for NRG2 is concentrated at the plasma membrane apposed to the centre of the SSC (Fig. 1e).

Next, we asked whether NRG2 puncta at SSCs represent the membrane-bound precursor (‘pro-NRG2’) or the proteolytically processed ectodomain. We began by labelling the ECD of NRG2 under non-permeabilizing conditions and the ICD after permeabilization. As shown in Fig. 1g, ECD and ICD patterns overlapped extensively, suggesting that NRG2 protein is indeed unprocessed at these sites. Moreover, sucrose density gradient fractionation of neocortical, hippocampal and cerebellar membranes revealed that the ∼110–120 kDa pro-NRG2 protein accumulated in the synaptosomal plasma membrane (SM) fraction, and that it was entirely resistant to extraction with 1% Triton X-100 (Fig. 1h,i). These data are consistent with the notion that pro-NRG2 accumulates in highly aggregated fashion near SSCs.

### NMDAR activity promotes NRG2 ectodomain shedding

Most NRGs are synthesized as membrane precursors that are converted to signalling-competent soluble ErbB receptor ligands via shedding of their ectodomains by ubiquitous alpha- or beta-secretases. To determine whether NRG2 is a suitable substrate for these extracellular proteases, we analysed PC12 cells that express very high levels of NRG2 mRNA but that accumulate very little pro-NRG2 at steady state (Supplementary Fig. 8). Indeed, inhibition of alpha-secretases by GM6001 (10 μM) triggered a robust increase of pro-NRG2 protein levels, thus unmasking constitutive shedding activity in this cell line.

**Figure 2:**
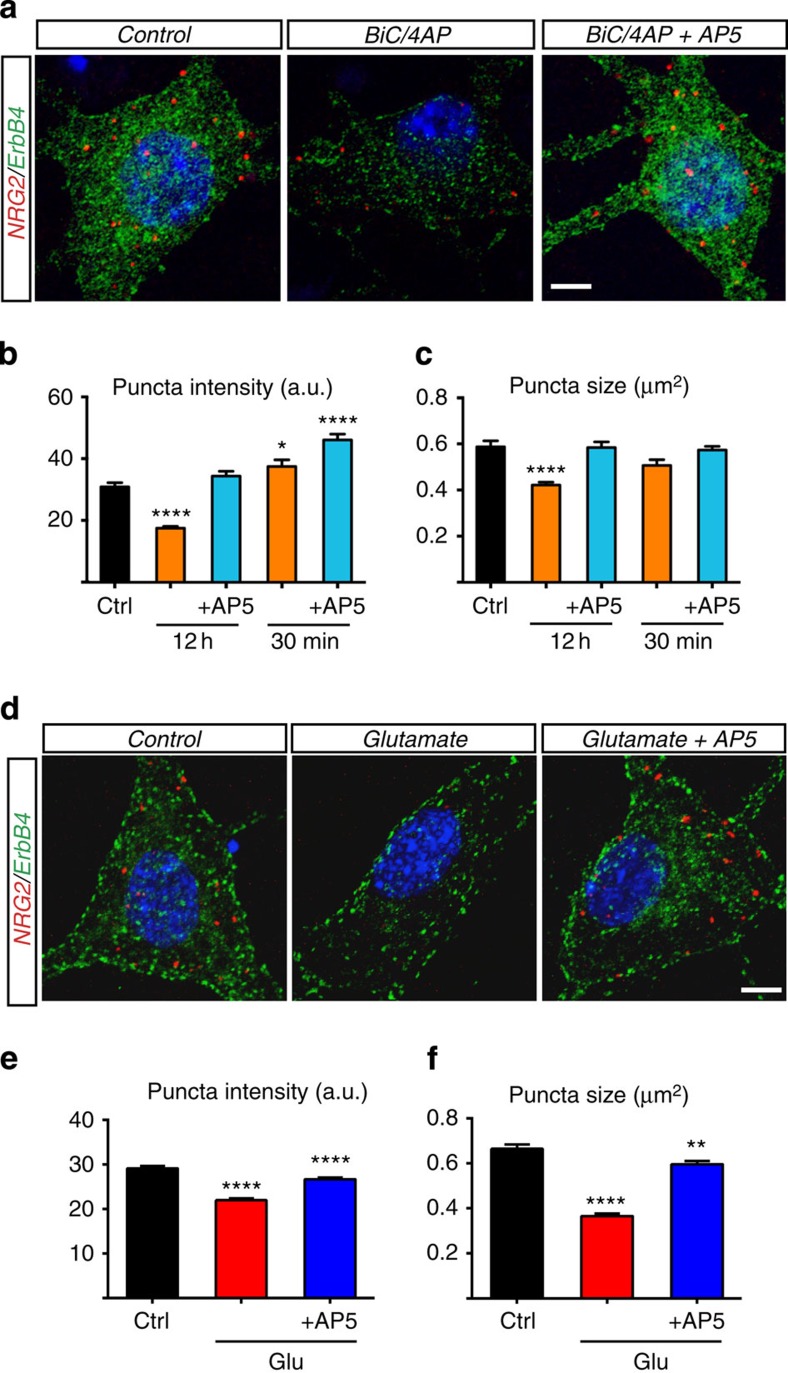
Activity and glutamate dynamically regulate NRG2 puncta. (**a**–**c**) Effect of chronic and acute burst firing on NRG2 puncta. (**a**) Representative confocal images of NRG2 puncta on cultured ErbB4^+^ interneurons in untreated controls, and after 12 h of Bic/4AP without or with the NMDAR inhibitor AP5. (**b**,**c**) Mean NRG2 puncta intensity (AU) and size (μm^2^) in ErbB4^+^ neurons. (**d**–**f**) Glutamate downregulates NRG2 puncta in an NMDAR-dependent manner. (**d**) Representative confocal images of NRG2 puncta on ErbB4^+^ interneurons, either untreated or after 10 min of 20 μM glutamate without or with 50 μM AP5. (**e**,**f**) Mean NRG2 puncta intensity (AU) and size (μm^2^) in ErbB4^+^ neurons. In both experiments, *n*=30 neurons from three independent experiments were analysed for each data point. Data represent the mean±s.e.m. *P<0.05; **P<0.01; ****P<0.0001 (one-way analysis of variance). Scale bars, 5 μm. Ctrl, control; Glu, glutamate.

We hypothesized that clustering of pro-NRG2 in neurons might serve to render proteolytic processing subject to activity-dependent regulation, as previously reported for NRG1 (refs 28, 29). We began by investigating the effects of elevating network activity on NRG2 puncta. Cultured hippocampal neurons were treated for 12 h with a combination of the GABA_A_ receptor blocker bicuculline (BiC; 50 μM) and the weak potassium channel blocker 4-aminopyridine (4AP; 250 μM), a treatment we refer to as BiC/4AP that is known to elicit chronic burst firing by disinhibiting network activity and engaging synaptic NMDARs^30^. As shown in Fig. 2a–c, 12 h of BiC/4AP treatment significantly decreased the mean NRG2 puncta intensity and size in ErbB4^+^ interneurons. The mean NRG2 puncta intensity decreased from 30.9±1.4 arbitrary units (a.u.) in untreated controls to 17.4±0.6 a.u. after 12 h of BiC/4AP treatment (Fig. 2b). The mean NRG2 puncta size was also reduced from 0.58±0.03 μm^2^ in controls to 0.42±0.01 μm^2^ (Fig. 2c). Importantly, BiC/4AP was without effect when the competitive NMDAR antagonist 2-amino-5-phosphonopentanoic acid (AP5; 50 μM) was present. Interestingly, when BiC/4AP was applied acutely (30 min), we observed a modest but significant increase in the mean puncta intensity to 37.5±2.2 a.u. that was further augmented by AP5 to 46.1±1.9 a.u.. Taken together, these results suggest that NRG2 puncta are in a dynamic equilibrium, with neuronal activity and NMDARs exerting opposing effects (see also Fig. 4a,b).

We then turned to more directly probe the involvement of glutamate, acting via NMDARs, to regulate NRG2. As shown in Fig. 2d–f, a 10-min treatment with non-cytotoxic concentrations of glutamate (20 μM) rapidly downregulated NRG2 puncta in an NMDAR-dependent manner. The mean puncta intensity decreased from 29.1±0.5 a.u. in untreated control cells to 21.9±0.4 a.u. with glutamate, an effect that was attenuated by AP5 (26.7±0.4 a.u.; Fig. 2e). The mean puncta size also decreased across all treatment groups (control: 0.66±0.02 μm^2^; glutamate: 0.36±0.01 μm^2^; glutamate+AP5: 0.59±0.01 μm^2^; Fig. 2f). Moreover, downregulation of NRG2 puncta by glutamate also manifested as a reduction of the mean number of puncta per cell (control: 17.3±2.5 versus glutamate: 5.8±0.9; Supplementary Fig. 9a) and the percentage of ErbB4^+^ interneurons with NRG2 puncta (control: 93.4%±0.1 versus glutamate: 56.6±1.9 versus glutamate+AP5: 89.6±5.7; Supplementary Fig. 9b). Interestingly, inclusion of AP5 significantly increased the mean number of puncta per cell (25.4±3.1), consistent with the idea of a dynamic equilibrium between NMDAR-independent processes that promote puncta formation, and NMDAR-dependent processes that promote puncta turnover.

**Figure 3:**
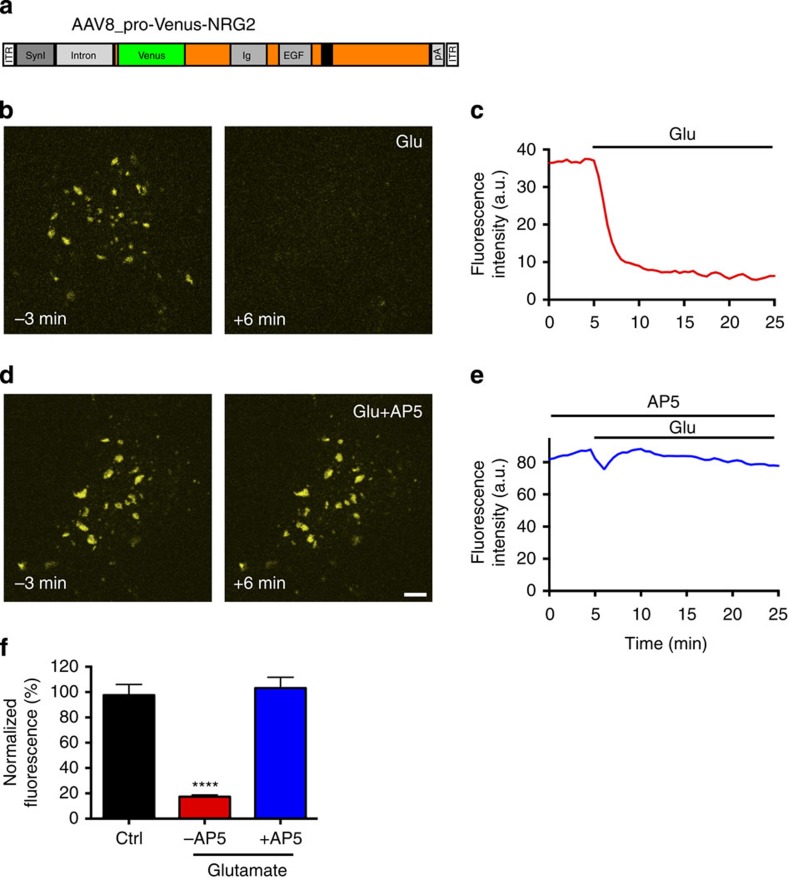
Analysis of glutamate-mediated NRG2 downregulation by live-cell imaging. (**a**) Schematic of the AAV construct to express Venus-NRG2 from the neuron-selective human Synapsin I promoter. (**b**,**d**) Representative images of live neurons expressing Venus-NRG2, 3 min before (left) and 6 min after the onset of 20 μM glutamate treatment in the absence (**b**) or presence of 50 μM AP5 (**d**). (**c**,**e**) Line graphs illustrating the time course of fluorescence intensities for neurons shown on the left. (**f**) Summary analysis of the effects of glutamate acting via NMDARs on Venus-NRG2 fluorescence. Fluorescence intensities are normalized to baseline (−3 min). *N*=17 (Ctrl), 18, (Glu) and 17 (Glu+AP5) neurons from three independent experiments. Data represent the mean±s.e.m. ****P<0.0001 (one-way analysis of variance). Scale bar, 5 μm. Ctrl, control; Glu, glutamate.

We next investigated whether glutamate promotes proteolytic processing of pro-NRG2 and the generation of a biologically active ectodomain. We were unable to detect endogenous NRG2 by western blotting in cultured neurons, likely owing to the relatively small number of NRG2-expressing cells and the sparse subcellular distribution of NRG2 puncta. We therefore generated an adeno-associated virus (AAV) construct with NRG2 amino-terminally fused to Venus fluorescent protein driven by the neuron-selective Synapsin I promoter (Fig. 3a). Clusters of Venus-NRG2 accumulated on cell bodies and proximal dendrites of infected neurons, but were typically larger in size than the puncta formed by endogenous NRG2. To verify that these clusters responded to glutamate, we monitored Venus fluorescence by live imaging before and after the addition of 20 μM glutamate. As shown in Fig. 3b,c,f, as well as the time-lapse movies available as Supplementary Movies 2 and 3, fluorescence intensities decreased rapidly following glutamate, fading to background levels within 2–5 min. As was the case for endogenous NRG2 puncta, this effect was dependent on NMDAR activity (Fig. 3d,e,f).

**Figure 4:**
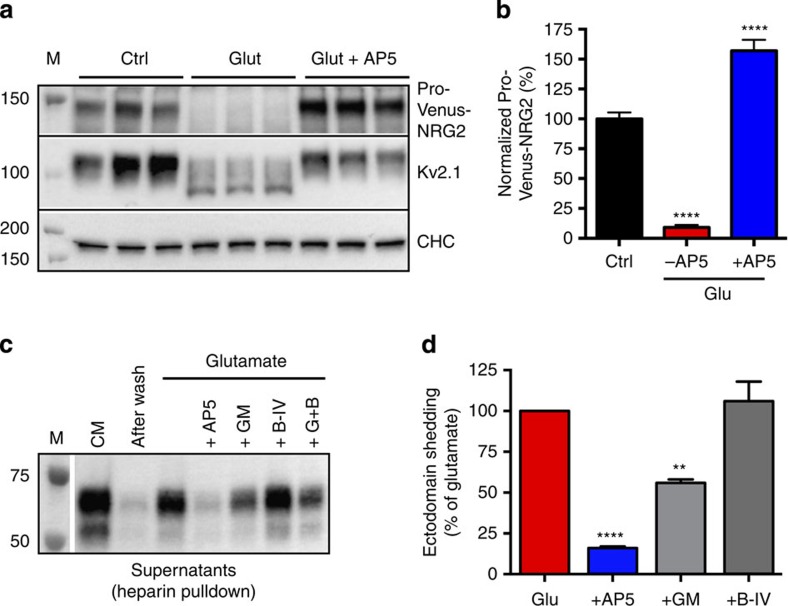
NMDAR activation triggers NRG2 ectodomain shedding. (**a**) Pro-Venus-NRG2 in whole-cell lysates of DIV 21 hippocampal neurons, stimulated for 20 min with 20 μM glutamate in the absence or presence of 50 μM AP5. Kv2.1 protein is shown to illustrate the characteristic downward shift in electrophoretic mobility in response to glutamate^37^. Clathrin heavy chain (CHC) is included as a loading control. (**b**) Quantitative analysis of pro-Venus-NRG2 downregulation by glutamate. Data are normalized to untreated controls. *N*=13 (Ctrl), 12 (Glu) and 8 (Glu+AP5) from six independent experiments. (**c**,**d**) Glutamate triggers NMDAR- and alpha-secretase-dependent NRG2 ectodomain shedding. Western blot in (**c**) shows Venus-NRG2 ectodomain protein in conditioned medium (CM), after replacement of CM with CM from uninfected cells, and after 20 min of 20 μM glutamate. Dependence of ectodomain shedding on NMDAR- and alpha-secretase activity revealed by pretreatment of cells with 50 μM AP5 and 10 μM GM6001, respectively. By contrast, ectodomain shedding was not affected by the inclusion of the beta-secretase inhibitor BACE-IV (1 μM). (**d**) Quantitative analysis of Venus-NRG2 ectodomain. Data are normalized to glutamate. *N*=3 independent experiments. Data represent the mean±s.e.m. **P<0.01; ****P<0.0001 (one-way analysis of variance). Ctrl, control; Glut (or Glu), glutamate.

Expression of pro-Venus-NRG2 was readily detected in cell lysates by western blotting using both anti-ECD and anti-ICD antibodies (Supplementary Fig. 10). In addition, two smaller bands representing the NRG2 ectodomain were observed with the ECD antibody in cell lysates and after capture of conditioned supernatant with heparin-agarose beads. This ectodomain had NRG activity, as demonstrated by the ability of purified conditioned medium to stimulate Erk2 phosphorylation in an ErbB receptor-dependent fashion in HEK293 cells (Supplementary Fig. 10). We then analysed Venus-NRG2 protein in hippocampal neurons treated for 20 min with glutamate. As shown in Fig. 4a and b, this treatment resulted in a ∼10-fold reduction of the pro-form (9.2±5.4% of control) and a concomitant increase in Venus-NRG2 ectodomain levels in the heparin-purified conditioned supernatants (Fig. 4c). Inclusion of AP5 not only prevented glutamate-augmented ectodomain shedding but also increased levels of the pro-form to 157±9.2% of control (Fig. 4b), consistent with the effects of AP5 on endogenous NRG2 puncta in cultures treated acutely with BiC/4AP (see also Fig. 2a–c). Moreover, pretreatment with GM6001 (10 μM), but not with the beta-secretase inhibitor BACE-IV (1 μM), significantly inhibited ectodomain shedding (glutamate+GM6001: 56±4% of glutamate; glutamate+BACE-IV: 106±10%), thus revealing the involvement of alpha- but not beta-secretases in NRG2 release downstream of NMDAR activation (Fig. 4c,d). In summary, we identified a novel NMDAR-dependent pathway that links glutamatergic signalling to the activation of NRG2 on ErbB4^+^ interneurons.

### NRG2 promotes the association of ErbB4 with GluN2B

**Figure 5:**
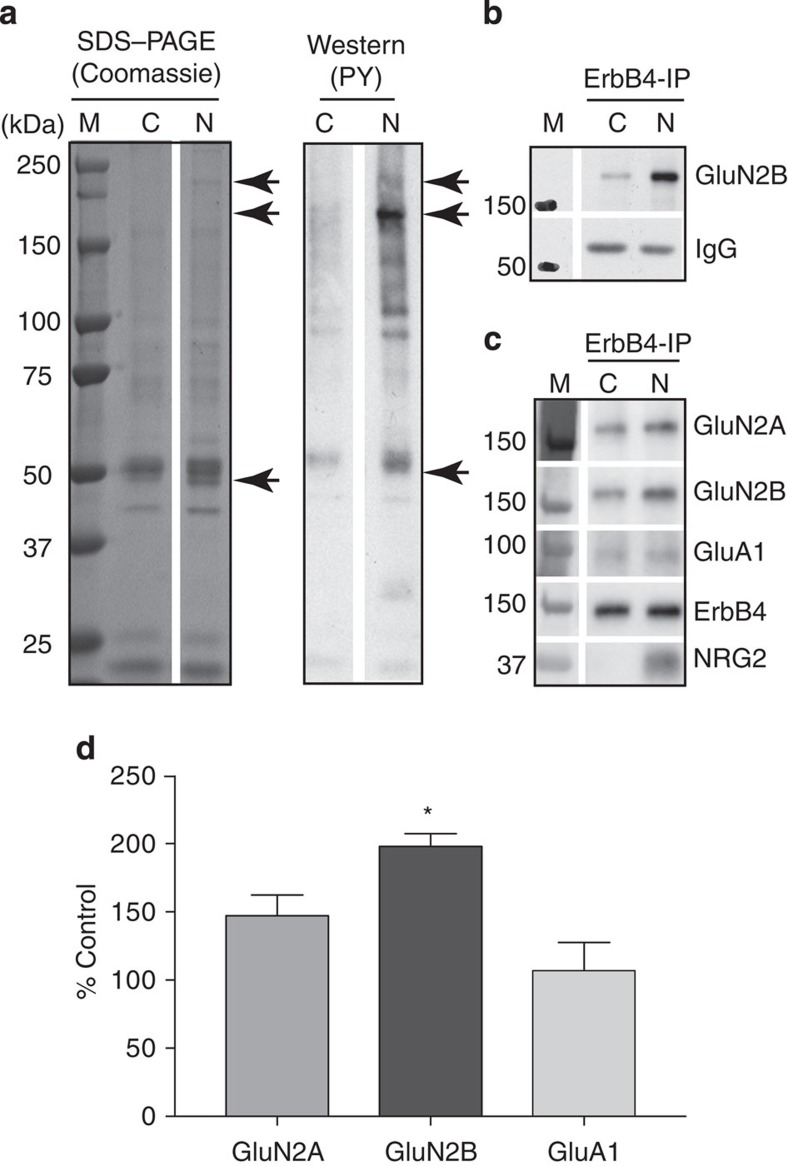
NRG2 promotes the association of ErbB4 with GluN2B-containing NMDARs. Proteins interacting with ErbB4 were isolated from metabolizing rat brain SMs following stimulation with 10 nM NRG2, and analysed by tandem MS (see Methods section for details). (**a**) Left, Coomassie-stained SDS–PAGE showing proteins obtained after ErbB4 immunoprecipitation from unstimulated control (C) and NRG2-stimulated membranes (N). Right, western blot of the same samples showing tyrosine-phosphorylated proteins. M, marker lane. Areas excised for MS are marked by arrows (see Supplementary Table 1 for a complete list of identified proteins). (**b**) ErbB4 stimulation by NRG2 (10 nM for 10 min) markedly increases association with GluN2B in metabolizing SMs from the rat cortex. IgG, antibody heavy chain signal. (**c**) NRG2 also promotes the association of ErbB4 with GluN2B, and to a lesser extent with GluN2A, in cultured hippocampal neurons (>DIV 28). Note that ErbB4 interactions with the AMPAR subunit GluA1 are not affected by NRG2. Western blot of NRG2 included to ascertain binding of ErbB4 in stimulated membranes. (**d**) Summary analysis of results shown in **c**. Data represent the mean±s.e.m. of densitometric signal ratios of glutamate receptor subunits over ErbB4, normalized to unstimulated controls (set as 100%). *N*=4 from three independent experiment; *P<0.05 (Student’s paired *t*-test).

We next used unbiased proteomics to identify possible direct targets of NRG2/ErbB4 signalling downstream of NMDAR activation. Metabolizing rat brain synaptosomes were treated for 10 min with 10 nM recombinant NRG2-ECD (hereafter referred to as NRG2), and ErbB4-interacting proteins were co-immunoprecipitated following TX-100 solubilization using the ErbB4 antibody mAB10 as described previously^16^. Coomassie-stained protein bands designated for mass spectrometry (MS) were selected based on western blots run in parallel to reveal NRG2-augmented phosphotyrosine signals (Fig. 5a). Using this approach, we unexpectedly identified three separate peptides (HSQLSDLYGK, DSVSGGGPCTNR and HGVVGGVPAPWEK) from the intracellular carboxyterminal tail region of the GluN2B subunit of the NMDAR, suggesting that the NMDAR itself is in a complex with ErbB4 (see Supplementary Table 1 for a complete list of all identified proteins). Subsequent direct western blot analysis of GluN2B protein in ErbB4 immunoprecipitates from metabolizing SMs (Fig. 5b), as well as from cultured neurons (Fig. 5c), confirmed a robust augmentation of ErbB4/GluN2B association following treatment with NRG2. In cultured neurons, NRG2 increased ErbB4-GluN2B co-immunoprecipitation to 198.1±9.3% of controls (Fig. 5d). ErbB4-GluN2A interactions were also modestly increased, although this effect did not reach statistical significance. Importantly, co-immunoprecipitation of ErbB4 with the α-amino-3-hydroxy-5-methyl-4-isoxazolepropionic acid receptor (AMPAR) subunit GluA1, abundantly expressed in GABAergic interneurons, was not altered by NRG2.

### NRG2 internalizes GluN2B-containing surface NMDARs

**Figure 6:**
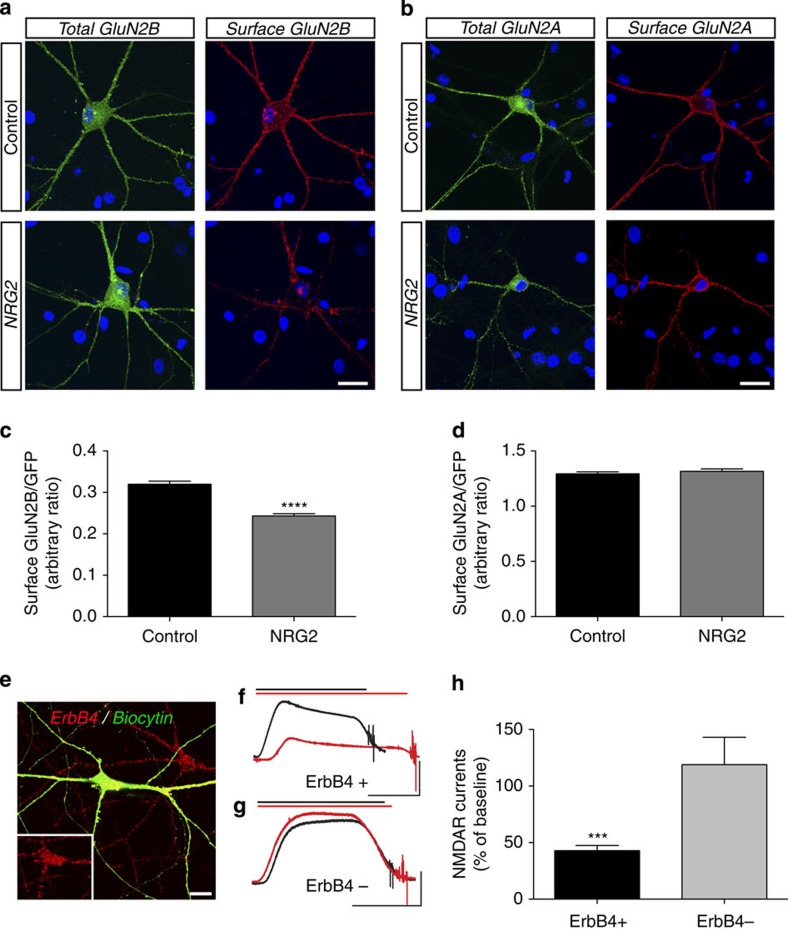
NRG2 promotes the internalization of GluN2B-containing NMDARs and the downregulation of whole-cell NMDAR currents in cultured ErbB4^+^ interneurons. (**a**–**d**) The effect of NRG2 on surface NMDAR expression was investigated in hippocampal neurons transfected with GFP-tagged GluN2A and GluN2B, and stimulated with NRG2 as described in Fig. 5. Surface NMDARs were labelled under non-permeabilizing conditions with anti-GFP (red), and surface+internal NMDARs were assessed using native GFP fluorescence (green). Cells were additionally labelled for ErbB4 (not shown) and nuclei were visualized with DAPI. (**a**,**b**) Representative images of total and surface GluN2B and GluN2A expression in vehicle and NRG2-stimulated ErbB4^+^ interneurons. Scale bars, 25 μm. (**c**,**d**) Quantitative analysis of relative surface expression of GFP-GluN2B (**c**) and GFP-GluN2A (**d**), expressed as arbitrary ratios of surface over total GFP fluorescence intensities. Data are mean±s.e.m. ****P<0.0001, *N*=6–12 neurons (Student’s *t*-test). (**e**–**h**) Effect of NRG2 (10 nM) on whole-cell NMDAR currents in >DIV 28 ErbB4^+^ and ErbB4^−^ hippocampal neurons. Currents were isolated by tetrodotoxin, 6-cyano-7-nitroquinoxaline-2,3-dione (CNQX) and picrotoxin at +40 mV in the presence of 100 μM NMDA delivered via a Y-tube. (**e**) Representative image of a recorded ErbB4^+^ interneuron identified by *post hoc* labelling. Scale bar, 25 μm. (**f**,**g**) Representative traces of whole-cell NMDA currents before (black), and after (red), 10 min perfusion with 10 nM NRG2 in ErbB4^+^ (**f**) and ErbB4^-^ neurons (**g**). Duration of NMDA treatments denoted by corresponding black and red horizontal bars. Note that the left and right tails of the NMDA-elicited currents mark the beginning and end of agonist delivery via the Y-tube; the jitter at the end of each trace reflects a perfusion artifact. Scale bars, 5 s and 500 pA. (**h**) Summary analysis of NRG2 effects on NMDA currents. Data are relative to baseline (set as 100%) and represent the mean±s.e.m. *N*=5 ErbB4^+^ and 4 ErbB4^−^ neurons; ***P<0.001% (Student’s *t*-test).

In cultured interneurons, acute treatment with recombinant NRG ligands has been linked to the reduction of currents and/or cell surface expression of voltage- and ligand-gated ion channels^14^,^15^,^16^. We therefore hypothesized that the physical interaction between activated ErbB4 and GluN2B might serve to downregulate surface GluN2B-containing NMDARs. To test this hypothesis, we transfected hippocampal neurons with plasmids expressing green fluorescent protein (GFP)-GluN2A and GFP-GluN2B. Cells were fixed and surface NMDARs were labelled under non-permeabilizing conditions using a GFP antibody. We then derived arbitrary ratios of GFP antibody-mediated fluorescence (surface) over native GFP fluorescence (total), and compared these ratios between untreated controls and cultures treated for 10 min with 10 nM NRG2. As shown in Fig. 6a–d, surface signals of ErbB4^+^ interneurons expressing GFP-GluN2B were significantly reduced after NRG2 (control: 0.320±0.007 a.u. versus NRG2: 0.243±0.005 a.u.). By contrast, relative surface levels of GFP-GluN2A were unaffected by NRG2 (control: 1.293±0.017 a.u. versus NRG2: 1.316±0.022 a.u.).

We then asked whether internalization of heterologously expressed GluN2B by NRG2 correlates with the downregulation of endogenous NMDAR function. We measured pharmacologically isolated whole-cell (synaptic+extrasynaptic) NMDAR currents in untransfected hippocampal neurons DIV 28 or older to ensure robust responses to NMDA. Recorded neurons were identified *post hoc* by ErbB4 immunofluorescence. We first measured baseline outward currents at +40 mV holding potential in response to 100 μM NMDA, and then again following 10 min of 10 nM NRG2 (both delivered via a Y-tube). As shown in Fig. 6e–h, whole-cell NMDA-evoked currents were significantly decreased in ErbB4^+^ interneurons after NRG2 treatment (43±4.5% of baseline). By contrast, NMDA-evoked currents in ErbB4-negative neurons were not affected by NRG2 (118±24.3% of baseline). Taken together, these findings demonstrate that NRG2-ErbB4 signalling downregulates NMDARs selectively in GABAergic interneurons.

### NRG2 selectively reduces evoked NMDAR EPSCs in interneurons.

**Figure 7:**
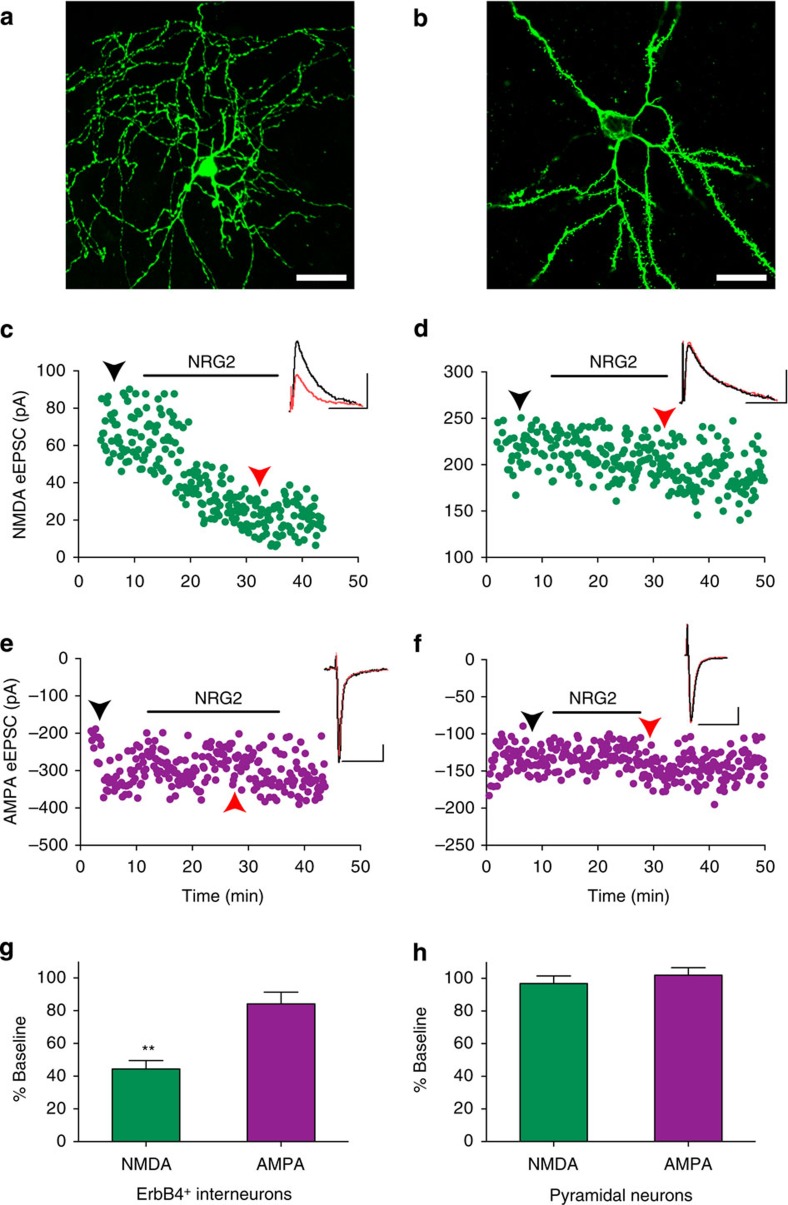
NRG2 acutely downregulates evoked NMDAR currents in cortical ErbB4^+^ interneurons but not pyramidal neurons. (**a**) Representative confocal image of an aspiny, biocytin-filled ErbB4^+^ interneuron from the PFC of an *Erbb4-2A-CreERT2 X Ai14* mouse used to record evoked NMDA and AMPA receptor currents. (**b**) Corresponding image of a biocytin-filled spiny PFC pyramidal neuron from a wild-type C57Bl/6J mouse. Scale bars, 25 μm. (**c**–**f**) Representative scatter plots of NMDA and AMPA receptor-mediated evoked excitatory postsynaptic currents (eEPSCs) recorded from an ErbB4^+^ interneuron (**c**,**e**) and from a pyramidal neuron (**d**,**f**). Duration of NRG2 treatment (10 nM) is indicated by horizontal bars. Arrows indicate the time points at which sample traces shown in the upper right corner were taken before (black) and after (red) NRG2. Scale bars in sample traces, 75 pA and 75 ms. (**g**,**h**) Summary plots of NRG2 effects on NMDA and AMPA eEPSCs in ErbB4^+^ interneurons (**g**) and pyramidal neurons (**h**). Data are normalized to baseline (set as 100%). *N*=6 (ErbB4^+^), 4 (pyramidal neurons). **P<0.01 (Student’s paired *t*-test).

We next analysed NRG2 effects on synaptic NMDARs. To this end, we recorded synaptically evoked NMDAR currents by whole-cell voltage-clamp in aspiny ErbB4-positive interneurons and spiny ErbB4-negative pyramidal neurons from acute mouse prefrontal cortical (PFC) slices before and after treatment with 10 nM NRG2 (Fig. 7a,b). ErbB4-positive interneurons were identified using *Erbb4_2A_CreERT2 x Ai14* mice^31^. Perfusion with NRG2 triggered a marked rundown of evoked NMDAR-mediated outward currents measured at +40 mV (Fig. 7c). After the onset of NRG2 treatment, normalized mean NMDAR EPSC amplitudes decreased to 44.4±5.2% of baseline (Fig. 7g). By contrast, synaptically evoked AMPAR inward currents measured at −70 mV were not significantly reduced (84.2±7.1%; Fig. 7e). Moreover, neither NMDAR nor AMPAR decay kinetics were altered, thus arguing against changes in receptor subunit composition. We also measured NMDAR- and AMPAR-mediated current responses to NRG2 in mPFC pyramidal neurons. Consistent with the notion that pyramidal neurons lack ErbB4, we failed to observe any effect of NRG2 on either glutamate receptor subtype (*n*=4; NMDA: 96.9±4.7%, *P*=0.37; AMPA: 102.0±4.5%, *P*=0.61; Fig. 7d,f,h).

## Discussion

In this study, we identify NRG2 as an important endogenous ErbB ligand that bidirectionally links NMDA and ErbB4 receptors in cortical GABAergic interneurons. We also introduce a number of novel concepts regarding activity-dependent regulation of NRG/ErbB4 signalling, and the nature of ErbB4-NMDA receptor interactions in the CNS. Namely, that ErbB4 and NRG2 form an autocrine signalling loop in inhibitory interneurons in which NMDAR activity is required to convert pro-NRG2 from a (presumably inactive) transmembrane precursor clustered at SSCs to a bioactive ErbB receptor ligand. In turn, NRG2 promotes the physical association of ErbB4 with GluN2B-containing NMDARs, which is followed by NMDAR removal from the cell surface and the downregulation of whole-cell and synaptic NMDAR currents selectively in interneurons. Taken together, these findings reveal an exquisitely close relationship between these two receptor systems that could serve as a new paradigm of how fast-acting neurotransmitter receptors and slow-acting neuromodulators interact in the regulation of GABAergic interneurons and cortical networks. Moreover, they are consistent with, and possibly provide the mechanistic underpinnings of, some of the apparent phenotypic similarities of engineered mice with interneuron-selective mutations of ErbB4 and NMDARs as they pertain to altered network synchrony—in particular increased gamma- and decreased theta-frequency oscillations—and the resulting effects on hippocampal and PFC local neuronal network activity^4^,^5^,^6^,^18^.

Earlier studies, using ISH probes targeting different NRG1 isoforms, reported that NRG1 is mostly expressed in cholinergic and glutamatergic neurons in the developing and adult brain^32^,^33^. To our knowledge, this is the first report of a neuregulin that is expressed in ErbB4^+^ cortical interneurons, and that accumulates as an unprocessed pro-form on the soma and proximal dendrites. This is in stark contrast to the distribution of CRD-NRG1, the major NRG1 isoform in the adult CNS and peripheral nervous system. In the hippocampus, CRD-NRG1 transcripts have been identified primarily in CA3 and the subiculum consistent with the expression in pyramidal neurons^34^, and multiple lines of evidence suggest that the CRD-NRG1 accumulates as a processed protein in axons and axon terminals^33^,^35^. We therefore propose that CRD-NRG1 and NRG2 engage in complementary signalling modes and thus subserve non-overlapping functions in the developed nervous system. However, it should be noted that unlike ErbB4, cortical NRG2 expression is not restricted to interneurons. Both autocrine and paracrine signalling are therefore likely to contribute to ErbB4 activation.

While the process that underlies the accumulation of pro-NRG2 at somatodendritic sites is not known, it is reasonable to speculate that the close spatial relationship to SSCs is a relevant factor. SSCs were originally described over 50 years ago^25^. Based on their ultrastructure and the presence of both ryanodine and InsP_3_ receptors, they have been likened to sarcolemmal triads and are believed to engage in calcium-dependent calcium release^36^. The accumulation of both NRG2 and Kv2.1 at SSCs, and their release from these sites in response to NMDAR activation (this work, ref. 37), suggests that the SSC and the associated plasma membrane form a signalling microdomain that dynamically regulates the subcellular distribution of these and possibly other proteins. Of note, electron-dense protein accumulations between SSCs and the adjacent plasma membrane were originally identified in cerebellar Purkinje cells^36^ that also prominently express somatodendritic NRG2 puncta. Interestingly, a report that appeared during review of the present work identified NRG1 clusters associated with SSCs in local cholinergic interneurons in the spinal chord^38^; however, we were unable to detect such clusters in cultured hippocampal neurons using the same NRG1 antibody. Notwithstanding, these findings suggest that association with SSCs might not be limited to NRG2.

**Figure 8:**
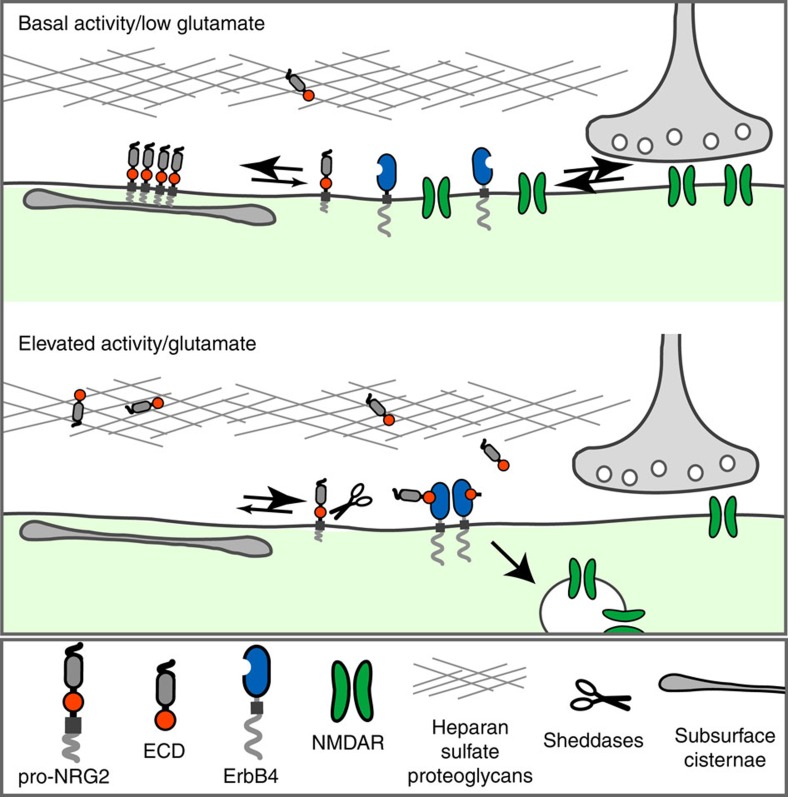
Reciprocal signalling between NMDA and NRG2/ErbB4 receptor systems. Working model illustrating the intricate relationship between NMDARs and NRG2/ErbB4 signalling in cortical interneurons. At baseline, low glutamate concentrations and weak NMDAR activity permit the accumulation of pro-NRG2 as highly concentrated clusters atop subsurface cisternae. Sudden increases in glutamate, acting via synaptic and/or extrasynaptic NMDARs, promote the rapid dispersal of NRG2 puncta and the shedding of the NRG2 ectodomain by extracellular proteases. The NRG2 ectodomain then accumulates locally by binding to heparan sulfate proteoglycans. Dissociation from the extracellular matrix, possibly aided by additional proteolytic processing events as suggested for NRG1 (ref. 58), enables NRG2 to bind its cognate receptor ErbB4. In this manner, autocrine NRG2/ErbB4 signalling initiates downstream events, including the internalization of GluN2B-containing NMDARs themselves. Based on the NRG2-dependent augmentation of ErbB4-GluN2B interactions in detergent-soluble cortical membranes fractions, surface NMDAR internalization experiments and our prior study on the regulation of GABAa1 receptor surface expression by NRG2/ErbB4 (ref. 16), we hypothesize that NMDAR internalization occurs primarily at extrasynaptic sites. Reduction of NMDAR EPSCs may result from a net loss of synaptic receptors due to lateral diffusion out of the synapse combined with removal of extrasynaptic/perisynaptic receptors.

Our analyses revealed surprisingly dynamic and bidirectional interactions between NMDARs and NRG2/ErbB4 signalling, as illustrated in our working model shown in Fig. 8. We favour a scenario in which NMDAR-mediated release of pro-NRG2 from highly clustered aggregates represents a critical first step, with subsequent ectodomain shedding by membrane-bound extracellular proteases depending largely on the availability of accessible substrate. This notion is supported by our finding that pro-NRG2 is constitutively processed in undifferentiated PC12 cells and thus does not depend on NMDAR activity for ectodomain shedding *per se*, and is consistent with current concepts of substrate selection by ubiquitously expressed sheddases^39^. Although we focused on the cell-autonomous nature of this process in interneurons, it should be noted that the signalling pathway downstream of NMDAR-mediated NRG2 ectodomain shedding almost certainly operates in other neuron types as well. This is based on the observation that hippocampal neurons infected with Venus-NRG2 responded consistently and uniformly to a glutamate challenge without any apparent effect of neuron type. Interestingly, although calcium-permeable AMPARs are abundant in cortical interneurons with low NMDAR content^40^, our pharmacological approach of pairing glutamate with AP5 strongly suggests that calcium-permeable AMPARs are not involved in glutamate-dependent NRG2 processing.

To our knowledge, this is the first study to report that NRG selectively downregulates NMDAR activity in ErbB4-expressing cortical and hippocampal GABAergic interneurons, but not in ErbB4-negative pyramidal neurons. NMDARs join a growing number of ligand-gated^14^,^16^ and voltage-gated^15^ ion channels that are directly regulated by neuregulin in ErbB4-expressing interneurons. In fact, the ability to internalize surface channels in response to ligand binding is emerging as a common theme in neuronal ErbB4 biology. Our finding differs from previous reports that interpreted NRG/ErbB4 effects on NMDAR function or on morphology as occurring directly in pyramidal neurons (see refs 23, 41, 42), and is consistent with a growing consensus that ErbB4 is not expressed by hippocampal and neocortical pyramidal neurons^7^,^8^,^15^,^16^,^18^,^43^. Moreover, morphological and functional studies in mice with targeted receptor ablation in distinct neuron subtypes indicate that changes in pyramidal neurons are mediated indirectly via ErbB4 in inhibitory interneurons^43^. In addition, pyramidal neuron properties can be indirectly affected by downstream neuromodulator pathways regulated by NRG^11^,^44^ or by altered local circuits^18^,^43^,^45^,^46^. For these reasons, in order to understand how NRG2/ErbB4 and NMDAR bidirectional signalling directly affects neuronal networks and behaviours, it will be important in the future to develop the proper genetic tools to target NRG2 and NMDAR in distinct neuronal subtypes.

Genetic variants of the NRG/ErbB signalling pathway are associated with schizophrenia and its endophenotypes, as well as with neurological disorders with intellectual disabilities and cognitive deficits^19^,^47^,^48^. Analysis of schizophrenia ‘at risk’ polymorphisms associated with changes in NRG1 and ErbB4 mRNA levels, as well as a *de novo* microdeletion of *ERBB4*, suggest altered NRG/ErbB4 signalling can result in behavioural impairments resembling aspects of schizophrenia^44^,^49^. These findings are supported by behavioural studies in mice engineered to express reduced or increased levels of NRG1 or ErbB4 (ref. 19). Atypical antipsychotics targeting dopamine receptors show efficacy for the treatment of positive symptoms, but not for negative symptoms and cognitive impairments. Presently, there is intense focus on developing novel drugs that target distinct aspects of the glutamatergic system, particularly in PFC networks, with the ultimate goal of increasing NMDAR activity as a means to improve negative symptoms and cognitive function. This approach is based on extensive clinical and animal studies showing that NMDAR antagonists have psychomimetic properties and elicit negative symptoms and cognitive deficits typical of schizophrenia^1^,^2^,^50^. Moreover, subjects with anti-NMDAR encephalitis^51^, an autoimmune disorder with antibodies targeting GluN1 and causing receptor internalization^52^, have been misdiagnosed with first episode schizophrenia because subjects present psychosis, disorder thoughts and catatonic dyskinesias.

Paradoxically, a variety of structurally independent NMDAR antagonists have repeatedly been shown to increase PFC excitability in humans and animals^50^. A likely resolution to this paradox emerges from studies in freely moving rats showing that MK-801 selectively reduces the firing of fast-spiking GABAergic interneurons in the mPFC and thereby disinhibits pyramidal neurons^53^, thereby modifying excitation/inhibition balance and cognitive (persevering) behaviours. In addition, NMDAR hypofunction at synaptic inputs onto fast-spiking GABAergic interneurons are associated with impairment of cortical gamma oscillations in schizophrenia and regulated by NRG/ErbB4 signalling^3^,^44^. Hence, targeting NMDARs selectively on GABAergic interneurons, rather than globally, could have pharmacological benefits for cognitive processes impaired in psychiatric disorders. Based on our findings that most ErbB4^+^ interneurons express NRG2, and that stimulation of ErbB4 with NRG2 causes the internalization of interneuron NMDARs, but not AMPARs, suggests that pharmacologically targeting NRG2 processing could have therapeutic value by selectively increasing NMDAR in GABAergic neurons.

## Methods

### Animals

*Erbb4-2A-Cre-ERT2* mice were provided by Dr Hongkui Zeng (Allen Institute for Brain Science, Seattle, WA), mice expressing tdTomato fluorescent protein from the Rosa26 locus (*Ai14*) and wild-type C57Bl/6J mice were obtained from the Jackson Laboratory^31^. Adult *Erbb4-2A-CreERT2 × Ai14* mice were injected intraperitoneally for three consecutive days with 1 mg tamoxifen (Sigma). Slices for electrophysiology were prepared 2 days after the last injection. Mice were kept on a 12–12-h light–dark schedule with access to food and water *ad libitum*. Animals were treated in accordance with the National Institutes of Health Animal Welfare guidelines. All procedures were approved by the NIH animal care and user committee.

### Drugs

NMDA (*N*-methyl-D-aspartatic acid), AP5, picrotoxin, BiC, CNQX, tetrodotoxin and 4AP were from Tocris. Glutamate was obtained from Sigma. GM6001, L-685,458 and BACE-IV were obtained from Calbiochem.

### NRG2-ECD

The sequence encoding the ECD (amino acids 20 through 313, based on the annotation in ref. 24) was cloned into a bacmid vector and flanked at the 5′-end by a gp67 signal peptide sequence and at the 3′-end by a hexahistidine tag sequence. Recombinant NRG2-ECD was purified by metal affinity chromatography from 5 l of insect cell medium conditioned by Sf9 cells infected with NRG2-ECD baculovirus (Genscript). Protein integrity and function were validated by SDS–polyacrylamide gel electrophoresis (SDS–PAGE), western blotting and by assaying ErbB receptor-dependent Erk pathway activation in HEK293 cells American Tissue Culture Collection (ATCC).

### NRG2 antibodies

Baculovirus/Sf9-derived NRG2-ECD was used as an immunogen to develop mouse monoclonal anti-NRG2 antibody 8D11 (hybridomas generated at Precision Antibodies) and rabbit polyclonal antiserum 7215 (Covance Research Products). An *Escherichia coli*-derived glutathione-S-transferase (GST) fusion protein harbouring 128 amino acids of the ICD of mouse NRG2β (residues 519 through 646) was used to develop rabbit monoclonal antibody mAB11 (hybridomas generated at Epitomics). Rabbit polyclonal antibody 1349 against the carboxyl terminus of mouse/rat NRG2 has been described elsewhere^20^. Polyclonal antibodies 7215 and 1349 were affinity purified against their respective immunogens. Monoclonal antibodies 8D11 and mAB11 were used as conditioned hybridoma supernatants or as Protein A-purified gamma-Ig (IgG) fractions. All antibodies were tested for their utility in western blotting, immunohistochemistry and immunofluorescence cytochemistry (see Supplementary Fig. 2 for additional information). In addition, the ECD antibody 7215 was validated for use as a neutralizing antibody. All NRG2 antibodies were used at 1 μg ml^−1^.

### AAV_Venus-NRG2

AAV (capsid serotype 8) harbouring the open reading frame of human hNRG2β was generated by Gateway-mediated recombination of entry vector pENTR223.1-hNRG2β (IMAGE clone 100066341) with Gateway-converted destination vector pAAV-MCS (Stratagene). For live-imaging experiments and western blotting, Venus fluorescent protein was inserted into the ECD of human hNRG2β immediately downstream of the signal sequence, and the fusion protein was expressed from the human Synapsin I promoter. AAV preparations were purified by iodixanol gradient centrifugation (Vector Development Core at Loyola University Chicago).

### Other antibodies

In addition to the NRG2 antibodies described above, commercial and custom antibodies against the following proteins were used in this study: ErbB4, rabbit monoclonal mAB10 (1 μg ml^−1^; ref. 7) and rabbit polyclonal custom antibody 5721 (unpublished; 0.4 μg ml^−1^); Kv2.1, mouse monoclonal (clone K89/34; NeuroMab; 1 μg ml^−1^); rabbit polyclonal antibody against NRG1 (SC-348; Santa Cruz Biotechnology; 0.2 μg ml^−1^); anti-V5 epitope tag (AbD Serotech; 1 μg ml^−1^); Bassoon, rabbit polyclonal (Synaptic Systems; 1:1,000); Gephyrin, mouse monoclonal (clone mAb7a; Synaptic Systems; 1:500); PSD95, mouse monoclonal (clone 7E3-1B8; Pierce; 1:500); Calbindin, mouse monoclonal (clone CB-955; Sigma; 1:1,000); GFP, rabbit polyclonal (Molecular Probes; 1:2,000); Erk2, rabbit polyclonal (C-14; Santa Cruz; 0.2 μg ml^−1^); phospho-Erk2, mouse monoclonal (clone E-4; Santa Cruz; 0.2 μg ml^−1^); GAPDH, mouse monoclonal (clone 6C5; Santa Cruz; 0.2 μg ml^−1^); clathrin heavy chain, mouse monoclonal (SC-12,734; Santa Cruz; 0.1 μg ml^−1^); GluN2A, rabbit monoclonal (clone A12W; Millipore; 1:2,000); GluN2B (clone 13; BD Biosciences; 1 μg ml^−1^); and GluA1 (AB1504; Millipore; 0.2 μg ml^−1^).

### Double ISH

We used RNAscope, a fluorescence-based multiplexing ISH technique^54^, to analyse expression of NRG2 mRNA in ErbB4^+^ GABAergic interneurons. Briefly, 12 μm fresh-frozen sagittal sections from 4-week-old wild-type mice were hybridized at 40 °C for 2 h with specific probe sets for mouse NRG2 and ErbB4 or NRG2 and Gad67. Probes were as follows: NRG2, accession no. NM_001167891.1, probe region 324–1,270; ErbB4: NM_010154.1, probe region 361–1,257 and Gad67: NM_008077.4, probe region: 62–3,113. Specific hybridization signals were amplified following the manufacturer’s instructions, and detected with Alexa 488 (for ErbB4 and Gad67) and Atto 550 (for NRG2) fluorophores. Sections were additionally labelled with the nuclear stain DAPI (4′,6-diamidino-2-phenylindole). Images were acquired using a LSM780 confocal microscope at × 63 magnification (Zeiss) and adjusted for overall brightness and contrast using ImageJ software.

### Immunohistology

Mice were transcardially perfused with 4% paraformaldehyde (PFA) in 0.1 M phosphate-buffered saline (PBS), pH 7.4. Brains were postfixed overnight in the same fixative and 50 μm sections were cut on a vibratome. Sections were blocked in 20% normal donkey serum (NDS), 1% bovine serum albumin, 0.25% TX-100 in 0.1 M phosphate buffer (PB) for 1 h at room temperature (RT) and incubated with primary antibodies in 0.1 M PB with 2% NGS and 0.25% TX-100 (dilution buffer) for 24 h at +4 °C with gentle rocking. Slices were washed in 0.1 M PB with 0.25% TX-100 for at least 30 min before incubation with fluorophore-conjugated donkey secondary antibodies for 90 min at RT in dilution buffer. DAPI stain was included to label nuclei (Molecular Probes). After extensive washes in PBS with 0.25% TX-100, sections were mounted on gelatin-coated slides, dried and mounted in Mowiol-DABCO (1,4-diazabicyclo-[2,2,2]-octane). Fluorescence was analysed on a Zeiss LSM510 Meta confocal microscope (Zeiss Microimaging) at × 20 and × 40 magnifications. Images were adjusted for overall brightness and contrast in Adobe Photoshop (Adobe Systems).

### Rat hippocampal neuron cultures

Dissociated hippocampal neurons were prepared from E19 Sprague–Dawley rat pups and plated on poly-D-lysine coated plastic for western blotting, or on poly-D-lysine coated coverslips for live imaging and post-fixation immunofluorescence cytochemistry and immunogold electron microscopy. Neurons were maintained in defined neurobasal/B27 medium (Life Technologies), and half of the medium was changed once every week. For the analysis of NRG2 puncta, medium was not changed for at least a week before the date of the experiment, based on our observations that medium change can trigger transient downregulation of puncta.

### Validation of NRG2 antibody specificity in HEK293 cells

HEK293 cells were plated on poly-D-lysine-coated coverslips and transfected with expression vectors for human NRG2 or mouse NRG1 types I-III using Xtreme HP DNA transfection reagent (Roche). All NRG1 isoforms were of the β1a type and harboured an additional V5 epitope tag right upstream of the EGF-like domain. The next day, cells were fixed in fresh 4% PFA in PBS supplemented with 4% sucrose for 15 min at RT. All subsequent incubations were carried out at RT. After extensive washing in PBS, cells were blocked and permeabilized for 1 h in PBS supplemented with 10% normal donkey serum and 0.1% TX-100. Cells were then incubated with mouse monoclonal NRG2 antibody 8D11 and rabbit monoclonal NRG2 antibody mAB11 (both at 1 μg ml^−1^) for 1 h in antibody dilution buffer (PBS with 2% normal donkey serum and 0.1% TX-100). Primary NRG2 antibodies were visualized by secondary anti-mouse and anti-rabbit antibodies conjugated to Cy3 and Alexa 488, respectively. After washout of unbound secondary antibodies, cells transfected with NRG1 constructs were additionally incubated with mouse monoclonal anti-V5 tag for 1 h in antibody dilution buffer, followed by anti-mouse secondary antibody conjugated to Alexa 405. Nuclei were labelled with RedDot2 fluorescent dye (Biotium). Coverslips were mounted in Mowiol/DABCO mounting medium and imaged on a Zeiss LSM510 confocal microscope at × 63 magnification.

### Immunofluorescence cytochemistry of cultured neurons

Neurons grown on 12 mm coverslips were fixed in fresh 4% paraformaldehyde in PBS supplemented with 4% sucrose for 15 min at RT. After extensive washing with PBS, cells were blocked and permeabilized with 10% NGS/0.1% TX-100 in PBS. For surface labelling, blocking solution without detergent was used. Cells were incubated with primary antibody for several hours at RT or overnight at +4 °C with gentle rocking, and for 1 h with donkey or goat secondary antibodies conjugated to Alexa Fluor 488 and Cy3 fluorophores (Molecular Probes or Jackson ImmunoResearch). DAPI or RedDot2 (Biotium) stains were included in all experiments to label nuclei. Coverslips were again washed with PBS, dipped in water and mounted on slides using Mowiol/DABCO mounting medium. For gated stimulated emission depletion super-resolution microscopy, NRG2 immunoreactivity was visualized with anti-rabbit IgG conjugated to Oregon Green 488 (Molecular Probes) and Kv2.1 immunoreactivity was visualized with anti-mouse IgG conjugated to biotin (Sigma), followed by Atto 425-Streptavidin (Fluka). Images were acquired using a × 100 objective. Laser lines were 470 and 506 nm, and 592 nm for the depletion beam. Signals were acquired with a hybrid detector. Structured illumination fluorescence microscopy was performed using the Delta-vision OMX V4 imaging system in 3D-SIM mode (Applied Precision). Images were taken with an Olympus PlanApo N × 60 1.42 NA oil objective. The microscope was calibrated before experiments to calculate both the lateral and axial limits of image resolution under our experimental conditions. Raw images were processed and reconstructed in 3D using SoftWoRx software (Applied Precision).

### Morphometric analysis of NRG2 puncta

Experiments were performed using 3–4-week-old cultured hippocampal neurons. For BiC/4AP treatments^30^, neurons were treated for 30 min or 12 h with 50 μM BiC and 250 μM 4AP in the presence or absence of 50 μM AP5. For glutamate treatments, neurons were treated for 10 min with 20 μM glutamate without or with AP5. Neurons were then fixed and labelled for NRG2 and ErbB4. Images of NRG2 puncta on ErbB4^+^ interneurons were acquired at × 63 magnification using LSM780 (BiC/4AP) or LSM510 (glutamate) confocal microscopes and analysed with Volocity 6 (PerkinElmer). NRG2 puncta were filtered for pixel intensity and a minimum particle size of 0.18 μm^2^. NRG2 signals were analysed for intensity (a.u. based on 8-bit scale) and puncta size (μm^2^). For the glutamate experiment, the number of NRG2 puncta per cell and the number of ErbB4^+^ cells with detectable NRG2 puncta was also recorded. Data were plotted using Prism6 (GraphPad software) and analysed for statistical significance using one-way analysis of variance (ANOVA) with Bonferroni’s multiple comparisons test.

### Pre-embedding immunogold electron microscopy of NRG2 puncta

Fixed cells were washed and then blocked with 5% NGS with or without 0.1% saponin for 40–60 min. Samples were incubated with mouse monoclonal anti-NRG2 (clone 8D11) and secondary antibodies (Nanogold, Nanoprobes) for 1 h, fixed with 2% glutaraldehyde in PBS overnight, silver enhanced (HQ kit, Nanoprobes), treated with 0.2% osmium tetroxide in 0.1 M PB at pH 7.4 for 30 min, en bloc stained with 0.25% uranyl acetate in acetate buffer at pH 5.0 for 1 h, dehydrated in graded ethanols and embedded in epoxy resin. Thin sections were examined on a JEOL 1200 EX transmission electron microscope and images collected with a digital CCD camera (AMT XR-100, Adanced Microscopy Techniques).

### Live imaging of Venus-NRG2 in cultured neurons

Hippocampal neurons grown on 25 mm coverslips were infected at DIV 3 with AAV8_Venus-NRG2. Cultures were changed on DIV 9 to phenol red-free neurobasal/B27 medium and imaged on DIV 21 or DIV 22 using a Nikon Eclipse Ti spinning disk microscope equipped with a heated stage. Cells were transferred to an Attofluor cell chamber and maintained at 37 °C and 5% CO_2_ for the duration of the experiment. Up to six individual cells were imaged simultaneously, and single frames were acquired every 30 s. Glutamate (20 μM) was bath-applied after recording a stable baseline for 5 min, and images were acquired for an additional 20 min. In some experiments, cells were pre-treated with AP5 (50 μM) during baseline acquisition. Following background subtraction, fluorescence intensities were quantified from regions of interest using Volocity software, and plotted against time using a sliding average of three frames. For summary analyses, fluorescence intensities were compared 3 min before and 6 min after the onset of treatment. Data were analysed using Prism6 (GraphPad software), normalized to baseline and tested for significance using one-way analysis of variance.

### Analysis of NMDAR surface expression

Dissociated hippocampal neurons grown on 12 mm glass coverslips were transfected on DIV 10 with plasmids encoding GFP-GluN2A or GFP-GluN2B (ref. 55) and treated at >DIV 21 with NRG2 (10 nM) for 10 min. Cells were fixed in 4% PFA in PBS containing 4% sucrose at RT for 15 min and blocked with 10% donkey serum in PBS for 1 h. For surface labelling, cells were incubated with anti-GFP antibody in blocking buffer for 3 h. Cells were washed several times with PBS and permeabilized with blocking buffer containing 0.1% TX-100. Cells were then incubated with rabbit polyclonal antibody 5721 against ErbB4. Following extensive washing with PBS containing 0.1% TX-100, cells were incubated with fluorescently labelled secondary antibodies for 45 min, washed and mounted in Mowiol/DABCO mounting medium.

Laser-scanning confocal images were acquired using an LSM510 microscope (Zeiss) and analysed using Volocity software. To quantify surface NMDARs, transfected cell were selected using the ‘find objects’ function on the GFP channel with a threshold filter of two s.d.’s and a minimum particle size of 0.18 μm^2^. Surfaces outside ErbB4-expressing cells were selected similarly and combined using the ‘exclude non-touching’ function (that is, GFP not touching ErbB4), resulting in surfaces that expressed GFP-NMDAR constructs and that were confined to ErbB4^+^ cells. Raw data were exported to Excel and the ratio of surface GFP over total GFP was calculated. Data were processed for statistical analysis using GraphPad Prism 5.0, and plotted as mean intensities.

### NRG2 ectodomain purification from conditioned medium

Conditioned medium was obtained from DIV 21 hippocampal neurons infected with Venus-NRG2 at DIV 3, and the NRG2 ectodomain was captured using heparin-agarose beads (Sigma). Beads were first equilibrated with cold neurobasal/B27 medium, and then incubated for several hours at +4 °C with conditioned medium. Protein bound to heparin was eluted with 500 mM NaCl following three washes with cold PBS. To analyse NRG2 ectodomain shedding in response to glutamate, infected cells were first washed into conditioned medium from age-matched uninfected cultures to reduce baseline NRG2-ECD levels, and heparin-associated proteins were eluted with SDS–PAGE sample buffer.

### Brain membrane fractionation

Brain tissue was dissected and quickly homogenized in 10–20 volumes cold 0.32 M sucrose/10 mM HEPES pH 7.4 using a glass-teflon homogenizer rotating at 900 r.p.m. or a hand-operated glass-glass homogenizer (for metabolizing synaptosomes). Nuclei and unbroken cells were spun out for 10 min at 1,000*g*. The supernatant S1 was centrifuged for 30 min at 17,000*g* to obtain the crude membrane pellet P2. Following hypoosmotic shock to release synaptic vesicles, the resulting P3 fraction was loaded onto a discontinuous sucrose gradient (0.32 M/0.8 M/1.0 M/1.2 M) in 10 mM HEPES pH 7.4 and centrifuged for 2 h at +4 °C and 150,000*g* using a SW60Ti rotor (Beckman). The 1.0/1.2 M sucrose interphase containing SMs was collected and extracted with 1% TX-100 to obtain detergent-soluble and detergent-insoluble subfractions. All solutions were supplemented with protease inhibitors (Roche). P2 fractions from whole rat brain were used as metabolizing SMs for NRG2 stimulation experiments.

### ErbB4 immunoprecipitation

NRG2- and vehicle-treated P2 SMs were solubilized with 1% TX-100 and incubated for 30 min at 4 °C with gentle agitation. Soluble and insoluble fractions were separated by centrifugation at 32,000*g* for 20 min. Soluble protein was adjusted to 1 mg ml^−1^ by addition of Ringer solution with 1% TX-100, protease inhibitors and NaVO_4_. Equal amounts of protein were pre-cleared with 20 μl per mg Protein A-agarose (Pierce) for 1 h at 4 °C with agitation. ErbB4 was immunoprecipitated with 10 μg per mg protein of rabbit monoclonal antibody mAb-10^7^ crosslinked to Protein A with 100 μM disuccinimidyl suberate (Pierce). For immunoprecipitation from hippocampal cultures, two wells of a six-well plate or 1–10 cm dish was used per condition. Following treatments, cultures were washed three times on ice with cold PBS and then lysed in PBS+ 1% TX-100 with protease and phosphatase inhibitors. Following a 30-min incubation at 4 °C, lysates were cleared at 5,000*g* for 5 min. Protein was adjusted to equal concentrations and ErbB4 was immunoprecipitated as above.

### MS of ErbB4-interacting proteins

ErbB4 immunoprecipitates from controls and NRG2-treated metabolizing synaptosomes were size-fractionated by SDS–PAGE, and Coomassie-stained gel bands were manually excised. In-gel tryptic digestion and peptide extraction were performed using a standard protocol^56^, followed by 1D liquid chromatography tandem MS (1D-LC-MS/MS) analysis using a Nano LC 1D Proteomics HPLC system (Eksigent) coupled online to an (LTQ)-Orbitrap mass spectrometer (Thermo Fisher) equipped with a Nanomate nanoelectrospray ionization source (Advion). 1D-LC-MS/MS experiments for protein pulldowns were performed such that spectra were acquired for 60 min in the data-dependent mode with dynamic exclusion enabled. The top five peaks in the 400–2,000 *m/z* range of every MS survey scan were fragmented. Survey spectra were acquired with 60,000 resolution in the Orbi-mass analyser and fragmented in the LTQ ion trap. 1D-LC-MS/MS experiments for peptide pulldowns were performed in a similar manner except the top three peaks in the 400–2,000 *m/z* range of every MS survey scan were fragmented, and survey spectra were acquired with 30,000 resolution.

### Western blotting

Protein samples from tissue lysates, cultured cells, conditioned medium or ErbB4 immunoprecipitates were size fractionated on 7.5% or 4–15% Mini-Protean TGX precast gels (Bio-Rad) and electroblotted onto nitrocellulose. Membranes were blocked with 3% bovine serum albumin in Tris-buffered saline (TBS) containing 0.1% Tween-20 (TBS/T), and incubated with primary antibodies in blocking solution for several hours at RT or overnight at 4 °C. After several washes with TBS/T, membranes were incubated with horseradish peroxidase-conjugated secondary antibodies (Jackson ImmunoResearch) for 1 h at RT. Signals were detected by chemiluminescence using a ChemiDoc MP imager (Bio-Rad) and quantified using Image Lab software (Bio-Rad). Full blots for all western blots shown in the figures are shown in Supplementary Fig.11.

### Whole-cell NMDAR currents

Hippocampal neurons grown on glass coverslips were transferred to a submerged recording chamber continuously perfused at 2 ml min^−1^ at 30–32 °C with artificial cerebrospinal fluid (ACSF) containing (in mM) 124 NaCl, 25 Na_2_HCO_3_, 11 glucose, 2.5 KCl, 1.3 MgCl_2_, 2.5 CaCl_2_ and 1.25 NaH_2_PO_4_ buffered via continuous bubbling with carbogen. Currents were low-pass filtered at 2 kHz and digitized at 1 kHz using Axopatch 200B amplifier, Digidata 1440A data acquisition boards, and pCLAMP10 software (all from Molecular Devices). Access resistance was monitored during recordings and experiments with >20% change were discarded. Whole-cell voltage clamp recordings of NMDA-evoked currents were performed with borosilicate glass microelectrodes (3–5 MΩ) filled with internal solution containing (in mM) 130 CsMeSO_3_, 8 NaCl, 10 HEPES, 0.5 EGTA, 4 Mg-ATP, 0.3 Na-GTP, 1 BAPTA, 5 QX-314Cl, 10 phosphocreatine and 0.3% biocytin at pH 7.2 adjusted with KOH. Following acquisition of whole-cell configuration, neurons were depolarized to +40 mV to alleviate Mg blockade of NMDARs and allowed to recover for ∼5–7 min. Outward NMDA-mediated currents were isolated by bath application of tetrodotoxin (1 μM), 6-cyano-7-nitroquinoxaline-2,3-dione (10 μM) and picrotoxin (100 μM). Currents were evoked by 100 μM NMDA and confirmed with the NMDAR antagonist AP5 (25 μM). All drugs delivered by bath application were also delivered through the Y-tube with the exception of NMDA and NRG2 that where delivered only via the Y-tube^57^. Data were normalized to baseline and tested for significance using a paired student’s *t*-test. For *post hoc* analysis of ErbB4 expression, neurons were fixed following recordings and labelled with anti-ErbB4 antibody 5721, Alexa Fluor 488-Streptavidin and DAPI.

### Synaptic electrophysiological recordings in acute slices

PFC slices (300 μm) from tamoxifen-injected *ErbB4_2A_CreERT2 x Ai14* mice on a C57Bl/6J background (4–6 weeks of age) and C57Bl/6J wild-type mice of both sexes were used to record from ErbB4^+^ interneurons and pyramidal neurons, respectively. Coronal slices were prepared in ice-cold ACSF (in mM: 125 NaCl, 24 NaHCO_3_, 2.5 KCl, 1.25 NaH_2_PO_4_, 0.5 CaCl_2_, 5 MgCl_2_ and 10 glucose). Slices were incubated in a holding chamber at 32 °C for 1 h in ACSF saturated with 95% O_2_ and 5% CO_2_, and then transferred to 28 °C ACSF and kept for at least 1 h before recording. Neurons were recorded from the layer 2/3 of the medial PFC and stimulated in the same layer at a distance of ∼100 μm using a glass monopolar electrode filled with ACSF. Stimulation intensity was adjusted to 50% of maximal response. To measure NMDAR and AMPAR currents, pipettes were filled with an internal solution containing (in mM) 130 CsCH_3_SO_3_, 8 NaCl, 10 HEPES, 0.5 EGTA, 4 Mg-ATP, 0.3 Na-GTP and 10 phosphocreatine at pH 7.2. The internal solution additionally included biocytin for *post hoc* neuron identification. Pipette and whole-cell capacitance as well as series resistance were compensated by >85%. ErbB4^+^ interneurons were identified by the expression of red fluorescence. Peak synaptic AMPA currents were measured at −70 mV and NMDA currents were measured at +40 mV; measurements of the NMDA receptor component were calculated 50 ms following the stimulation artefact to minimize contributions from AMPA receptor currents^11^. The recording solution contained picrotoxin (100 μM) to block GABAR-mediated currents. After recording an initial baseline for 10–12 min, recombinant NRG2 (10 nM) was bath-applied until a stable response was attained (15–25 min). Command voltage and current stimulus were controlled by pClamp 10.1 via a digital/analogue interface (Digidata 1322A, Molecular Devices). The acquisition rate was 4 kHz and digitized at 20 kHz. Data were analysed using SigmaPlot (Systat Software), normalized to baseline and tested for significance using a paired Student’s *t*-test.

## Additional information

**How to cite this article:** Vullhorst, D. *et al.* A negative feedback loop controls NMDA receptor function in cortical interneurons via neuregulin 2/ErbB4 signalling. *Nat. Commun.* 6:7222 doi: 10.1038/ncomms8222 (2015).

## Supplementary Material

Supplementary Information Supplementary Figures 1-11, Supplementary Table 1 and Supplementary References

Supplementary Movie 1 3D-Reconstruction of NRG2 / Kv2.1 co-localization in a 2 month-old rat hippocampal neuron using structured illumination (SIM) microscopy. Image was acquired using the Delta-vision OMX V4 imaging system in 3D-SIM mode (see **Methods**). A stack of 37 z-sections was taken in steps of 125 nm to cover the entire cell body (4.5 μm). Raw images were processed and reconstructed in 3D using DV SoftWoRx software.

Supplementary Movie 2 Time-lapse of the NMDAR-dependent downregulation of Venus-NRG2 clusters in response to glutamate. Neurons were infected at DIV3 with Venus-NRG2 and live-imaged on DIV 21 (see **Methods**). The movie depicts representative neurons shown in **Figure 3**, with individual frames acquired every 30 seconds. Baseline fluorescence was acquired for 5 min, either in the absence (S2) or presence (S3) of 50 μM AP5, followed by bath application of 20 μM glutamate for 10 min. In movie S2, Venus-NRG2 clusters dim immediately after the addition of glutamate and quickly fade to background levels. In movie S3, Venus-NRG2 fluorescence remains stable throughout the duration of the recording session due to the blockade of NMDA receptors by AP5.

Supplementary Movie 3 Time-lapse of the NMDAR-dependent downregulation of Venus-NRG2 clusters in response to glutamate. Neurons were infected at DIV3 with Venus-NRG2 and live-imaged on DIV 21 (see **Methods**). The movie depicts representative neurons shown in **Figure 3**, with individual frames acquired every 30 seconds. Baseline fluorescence was acquired for 5 min, either in the absence (S2) or presence (S3) of 50 μM AP5, followed by bath application of 20 μM glutamate for 10 min. In movie S2, Venus-NRG2 clusters dim immediately after the addition of glutamate and quickly fade to background levels. In movie S3, Venus-NRG2 fluorescence remains stable throughout the duration of the recording session due to the blockade of NMDA receptors by AP5.
