# Supplementary material for: A negative feedback loop controls NMDA receptor function in cortical interneurons via neuregulin 2/ErbB4 signalling
Source: Nat Commun. 2015 Jun 1;6:7222. doi: 10.1038/ncomms8222 (PMC4451617; doi:10.1038/ncomms8222)
Supplement: Supplementary Information — Supplementary Figures 1-11, Supplementary Table 1 and Supplementary References [file ncomms8222-s1.pdf]

A negative feedback loop controls NMDA receptor function in cortical interneurons via Neuregulin 2/ErbB4 signaling

Detlef Vullhorst, Robert M. Mitchell, Carolyn Keating, Swagata Roychowdhury, Irina Karavanova, Jung Hwa Tao-Cheng, Andres Buonanno

SUPPLEMENTARY INFORMATION

Includes:

11 Supplementary Figures

1 Supplementary Table

Supplementary References

SUPPLEMENTARY FIGURES

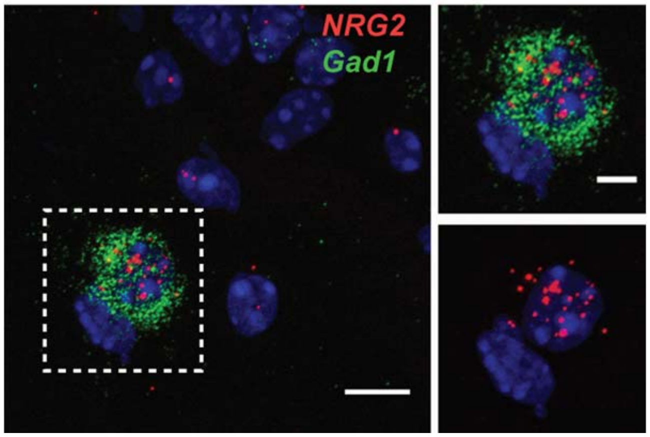

Supplementary Figure 1 NRG2 mRNA is expressed in hippocampal Gad1^{+} cells. Double immunofluorescence ISH of NRG2 and Gad1, showing overlapping signals in a neuron located in the stratum oriens of area CA1. DAPI was added to label nuclei (blue). Note that Gad1^{-} cells have much lower or no NRG2 signal. Micrograph is a z-projection of a stack of 10 confocal images. Boundary box on the left outlines the area magnified on the right. Scale bar = 10 µm (overview), 5 µm (magnified area).

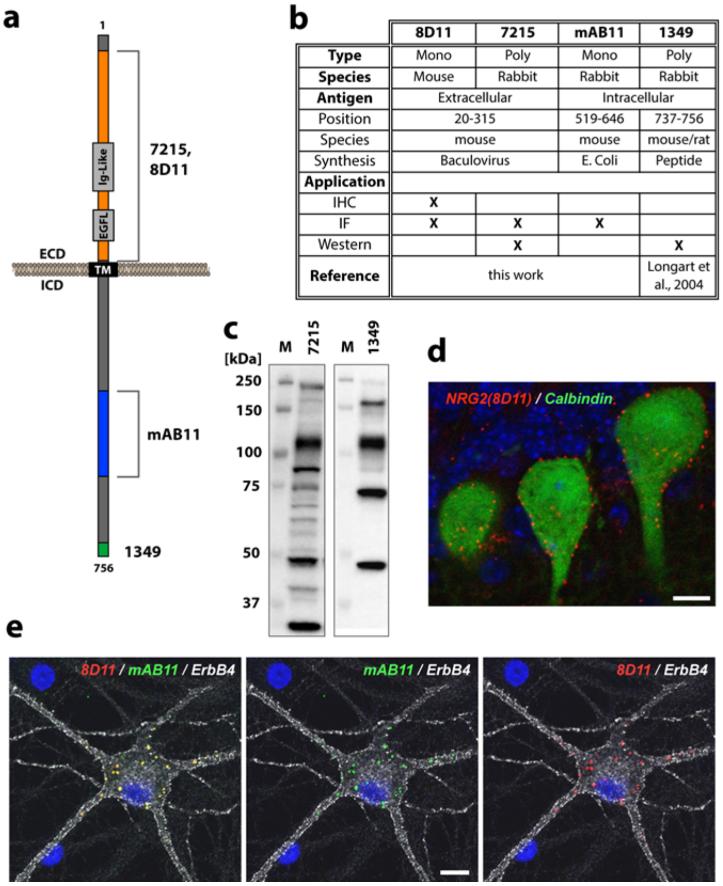

	8D11	7215	mAB11	1349
**Type**	Mono	Poly	Mono	Poly
**Species**	Mouse	Rabbit	Rabbit	Rabbit
**Antigen**	Extracellular		Intracellular	
Position	20-315		519-646	737-756
Species	mouse		mouse	mouse/rat
Synthesis	Baculovirus		E. Coli	Peptide
**Application**				
IHC	X			
IF	X	X	X	
Western		X		X
**Reference**	this work			Longart et al., 2004

Supplementary Figure 2 Generation and validation of NRG2 antibodies used for this study. (a) Map of immunogen sites on NRG2 used to raise poly- and monoclonal NRG2 antibodies. Amino acid annotations are based on [1]. *EGFL*, EGF-like domain; *TM*, transmembrane domain; *ECD*, extracellular domain; *ICD*, intracellular domain. (**b**) Overview of NRG2 antibodies, including information regarding validated applications. Note that no single antibody was found to perform optimally in Western blotting, immunofluorescence cytochemistry and immunofluorescence histochemistry. (**c**) Immunodetection of the ~110 kDa pro-NRG2 band (arrowhead) with rabbit polyclonal anti-ECD antibody 7215 and anti-ICD antibody 1349 in cerebellar P2 membrane fractions from adult mice. (**d**) Confocal microscopy images of cerebellar Purkinje cells labeled with antibodies against Calbindin and NRG2 (8D11), showing selective labeling of somatodendritic puncta. DAPI stain was added to label nuclei. (**e**) Triple labeling of a cultured DIV21 hippocampal ErbB4^{+} interneuron showing overlapping signals with NRG2 antibodies against the ECD (8D11) and ICD (mAB11). Scale bars = 10 µm.

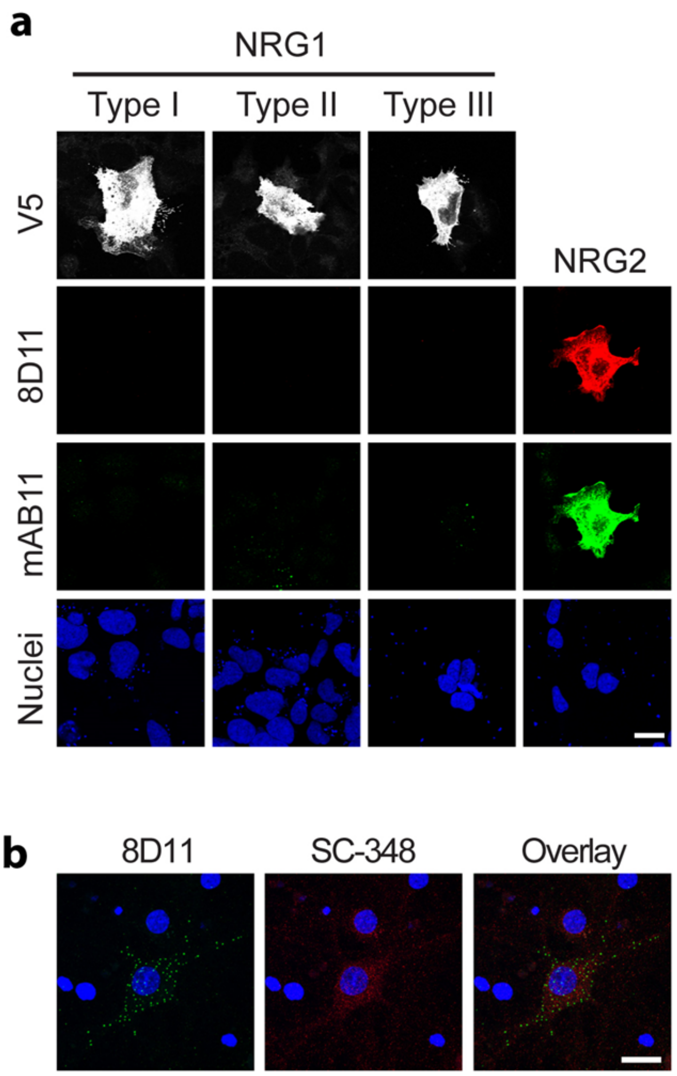

Supplementary Figure 3 Monoclonal NRG2 antibodies do not cross-react with NRG1. (**a**) Immunuflorescence cytochemistry of HEK293 cells transfected with plasmids encoding NRG1 types I-III and NRG2 using mouse monoclonal 8D11 and rabbit monoclonal mAB11. NRG1 constructs additionally harbored a V5 epitope tag for positive identification of transfected cells. Nuclei were labeled with RedDot2 and pseudocolored. (**b**) Double immunofluorescence cytochemistry of a cultured hippocampal neuron using anti-NRG2 ECD antibody 8D11 and anti-NRG1-ICD antibody SC-348. Note that the immunoreactivity obtained with SC-348 was generally low and diffuse, and lacked the somatodendritic signal clusters seen with NRG2 antibodies 8D11 and mAB11. Scale bars = 20 μm.

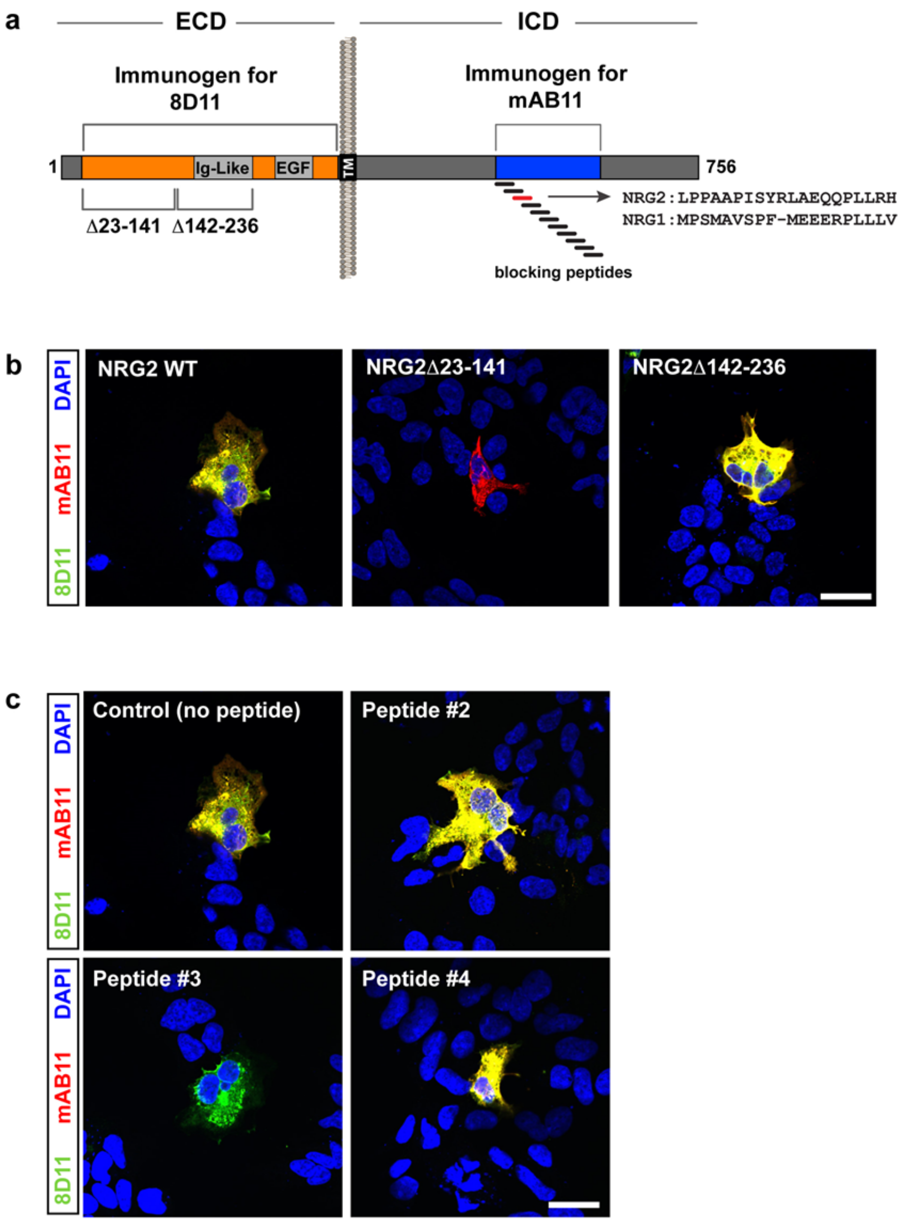

Supplementary Figure 4: Monoclonal antibodies against the ECD and ICD bind to unique sequences in NRG2 that are not conserved in NRG1. For mouse monoclonal 8D11, the binding region was mapped using two NRG2 constructs with deletions in the ECD. For rabbit polyclonal mAB11, the binding epitope was delineated using a peptide blocking approach. (***a***) Schematic illustration of NRG2 protein, with immunogen sequences for 8D11 in the ECD region and for mAB11 in the ICD region indicated. The areas deleted are shown below the ECD and encompass amino acids 23-141 (a region that is poorly conserved between NRG2 and NRG1 [1]),

and 142-236 that includes the conserved Ig-like domain. For the ICD, 11 overlapping peptides (20 amino acids each) corresponding to the aggregate sequence of the immunogen are shown. Peptide #3 is marked in red to indicate that only this peptide was capable of blocking the detection of NRG2 by mAB11 (see **c**), suggesting that the mAB11 epitope contains amino acids from the center region of this peptide not fully present in the flanking peptides. The sequence of this peptide and the corresponding sequence in NRG1 are shown (alignment based on Carraway et al. [1]). (***b***) Representative immunofluorescence images of 8D11 reactivity in HEK293 cells transiently transfected with plasmids expressing wild-type NRG2, NRG2Δ23-141 or NRG2Δ142-236. Cells were co-labeled with 8D11 (green) and mAB11 (red); images are overlays of signals derived from both channels. (***c***) Representative immunofluorescence images of mAB11 peptide blocking experiments. 1 μg of mAB11 was pre-incubated with 1 μg of blocking peptide for 1 hr at room temperature before immunofluorescence cytochemistry of NRG2 in transiently transfected HEK293 cells. Mouse monoclonal antibody 8D11 against the ECD of NRG2 was included in all experiments, as was a control in which mAB11 was used without peptide. Images show the overlap of 8D11 (green) and mAB11 signals (red). Note that, like the control, peptides 2 and 4 except give rise to yellow overlay signal indicating binding of both antibodies to NRG2 while only 8D11 binding is with peptide #3. None of the other 8 peptides not shown here blocked mAB11. All images include DAPI to stain nuclei. Scale bar = 20 μm.

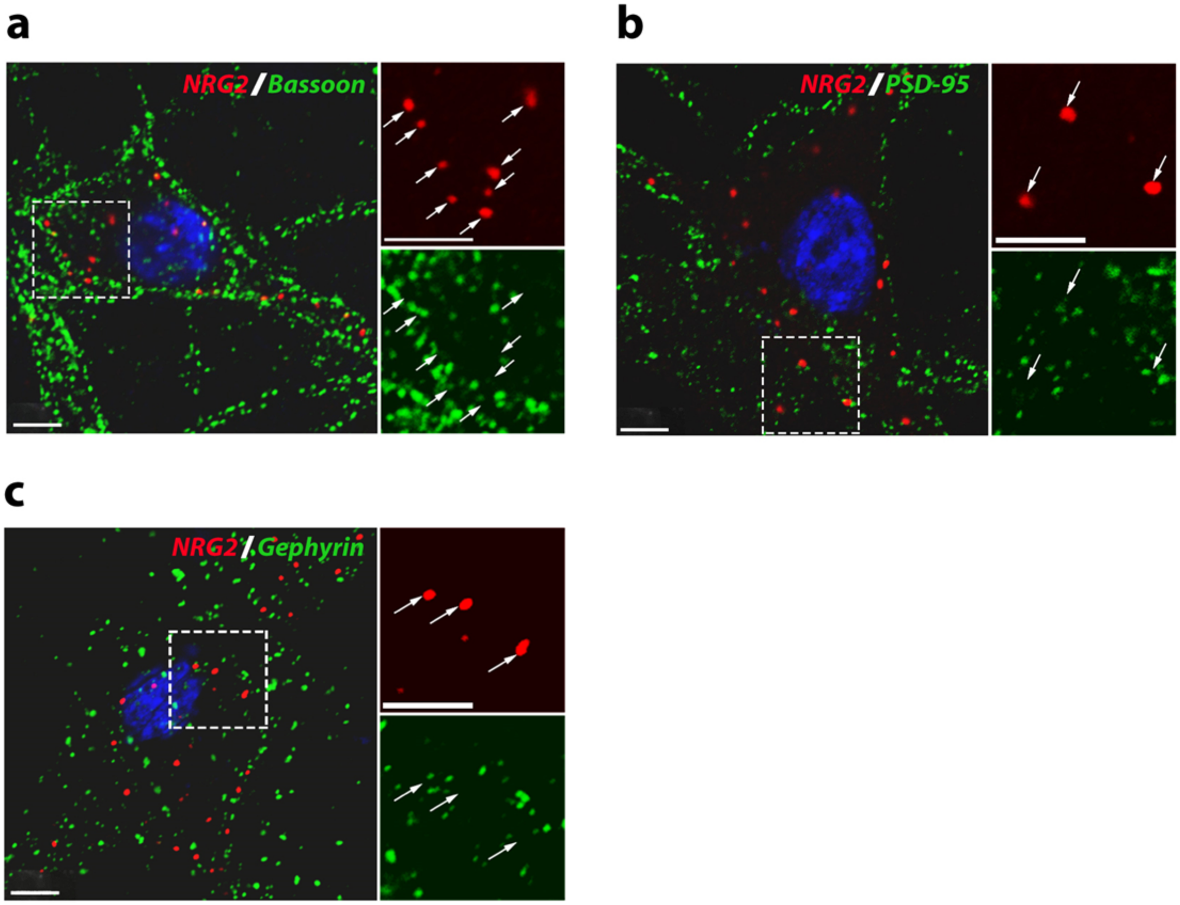

Supplementary Figure 5 NRG2 does not co-localize with synaptic markers. Two month-old hippocampal neurons were labeled for NRG2 and the presynaptic marker Bassoon (**a**), the postsynaptic glutamatergic scaffolding protein PSD-95 (**b**) and the postsynaptic GABAergic scaffolding protein Gephyrin (**c**). Arrows in magnified areas on the right indicate the location of NRG2 puncta. Note the absence of yellow in the overlay images on the left. Scale bar = 5 µm.

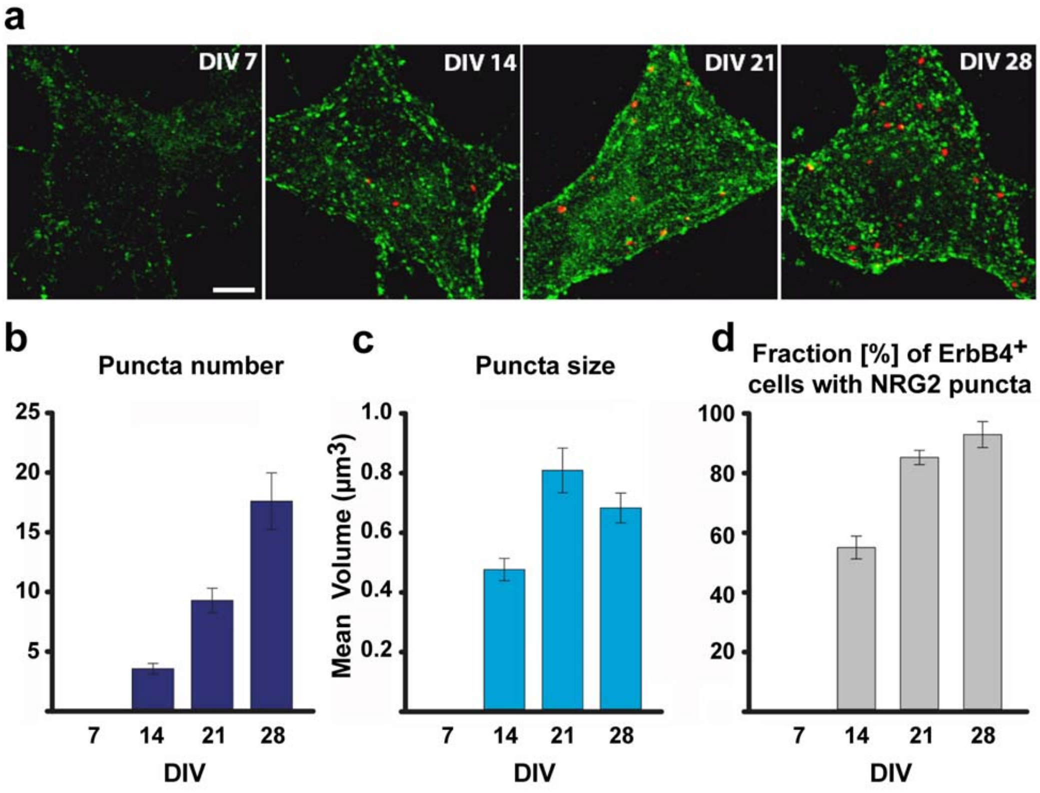

Supplementary Figure 6 Time course of the appearance of NRG2 puncta on cultured hippocampal ErbB4+ interneurons. Hippocampal neurons were fixed at indicated times and double-labeled for ErbB4 using mouse monoclonal antibody Ab-1 and for NRG2 using rabbit monoclonal antibody mAB11. (**a**) Representative examples of NRG2 immunoreactivity on $ErbB4^+$ interneurons at DIV7, DIV14, DIV21 and DIV28. Micrographs are projections of Z-stacks. Scale bar = 5 µm. (**b-d**) Quantitative analysis of NRG2 puncta formation. For each time point, Z-stacks of 21 images from 10 neurons were deconvolved using Volocity software and analyzed to derive the mean number of puncta per cell (**b**) and mean puncta volume (μm^3) (**c**). In addition, (**d**) shows the number of $ErbB4^+$ interneurons with NRG2 puncta for every time point. Data in **b-d** represent the mean ± S.E.M. (One-way ANOVA)

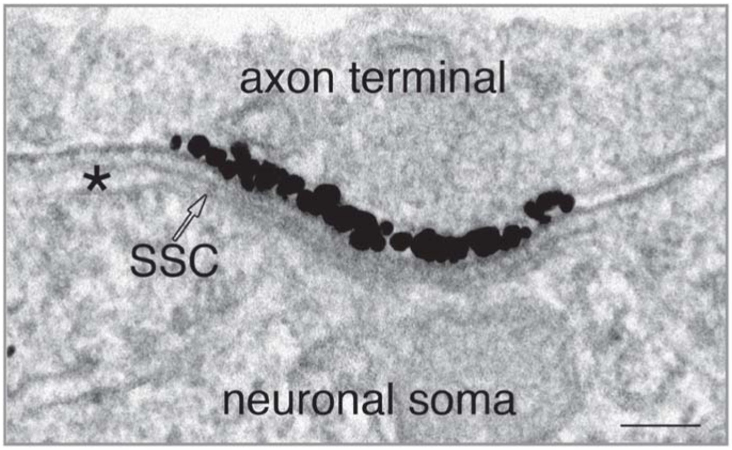

Supplementary Figure 7 Electron micrograph of label for NRG2 at the outer surface of the plasma membrane of a neuronal soma. The concentrated patch of signals is apposed to the flattened stack of subsurface cistern (SSC). Asterisks mark the open lumen of this SSC. This specialized area is apposed by an axon terminal filled with vesicles, a finding consistent with a previous report that SSCs can be apposed to presynaptic terminals [2]. Scale bar = 0.1 µm.

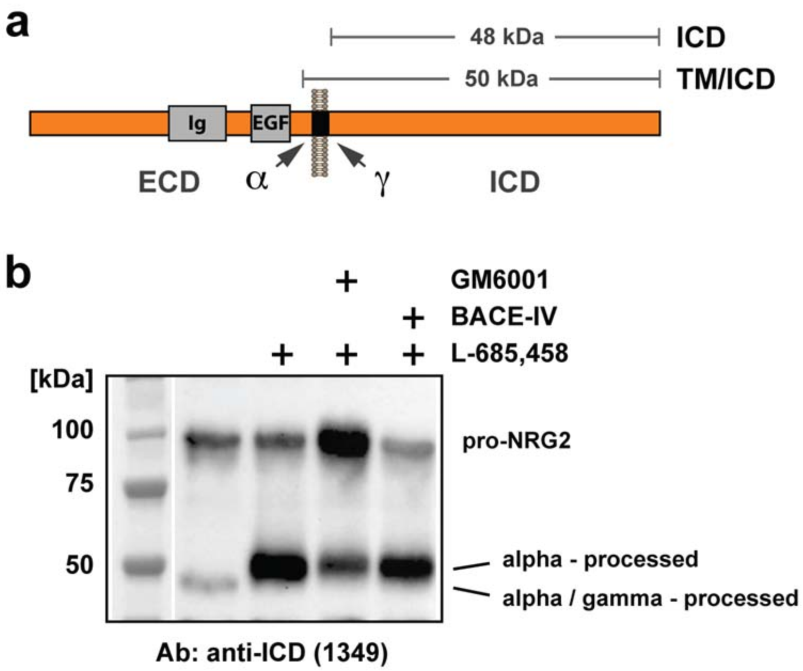

Supplementary Figure 8 Pro-NRG2 is constitutively processed by alpha and gamma secretases in PC12 cells. (**a**) Schematic illustration of NRG2 fragments generated by alpha- and gamma-secretases. Alpha-secretases cleave pro-NRG2 near the extracellular face of the transmembrane domain. Subsequent processing near the intracellular face of the transmembrane domain by gamma-secretase additionally releases a soluble intracellular fragment. Calculated molecular masses for the two proteolytic fragment identified by Western blotting in (b) are shown. (**b**) Regulation of NRG2 expression in whole PC12 cell lysates by alpha- and gamma secretases. Cells were either untreated or treated overnight with the gamma secretase inhibitor L-685,458 (1 μM) in the presence or absence of the alpha-secretase inhibitor GM6001 (10 μM) or the beta-secretase inhibitor BACE-IV (1 μM). The resulting Western blot was probed with anti-ICD antibody 1349. Alpha-secretase inhibition strongly augments the intensity of the pro-NRG2 band. Also note the shift in mobility of the NRG2 fragment detected in cells treated with L-685,458, consistent with the accumulation of the 50 kDa TM/ICD fragment as a result of gamma-secretase inhibition. The increased signal intensities of the TM/ICD fragment after L-685,458 treatment relative to the fully processed ICD in untreated PC12 cells suggest instability of the ICD. Beta-secretase does not appear to be involved in NRG2 ectodomain shedding in PC12 cells.

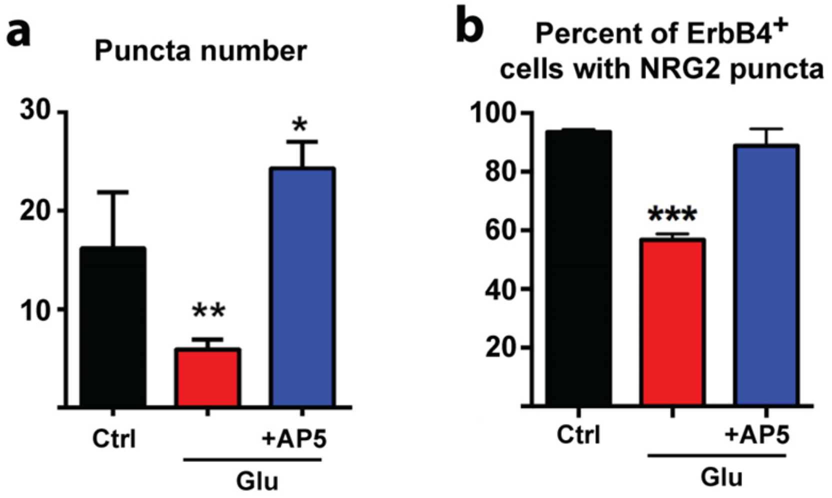

Supplementary Figure 9 Acute treatment with glutamate (20 µM, 10 min) strongly reduces the number of NRG2 puncta per cell (**a**) as well as the number of ErbB4+ interneurons with detectable NRG2 puncta (**b**). The glutamate effect is completely blocked by NMDAR blockade with AP5. N=30 neurons from 3 independent experiments. Data represent the mean ± S.E.M. *, $p<0.05$; **, $p<0.01$;***; $p<0.001$ (One-way ANOVA).

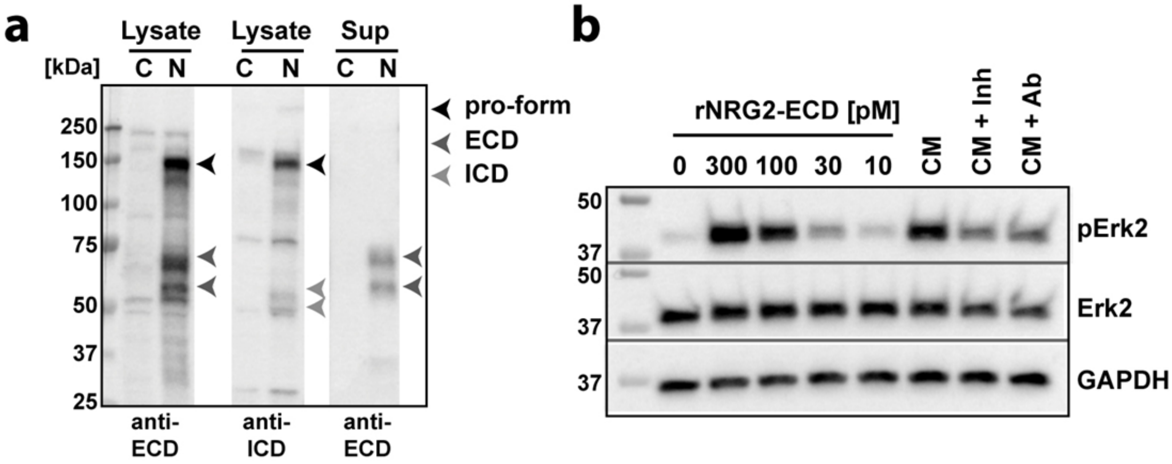

Supplementary Figure 10 Venus-NRG2 ectodomain purified from infected hippocampal neurons is biologically active. (**a**) Western blot analysis of lysates and heparin-purified supernatants from uninfected (C) and Venus-NRG2-infected (N) hippocampal neurons using ECD and ICD antibodies. Bands corresponding to pro-Venus-NRG2, as well as the corresponding ECD and ICD fragments, are marked by arrowheads. (**b**) Stimulation of Erk2 kinase activity in HEK293 cells using heparin-purified conditioned medium (CM) from Venus-NRG2-infected hippocampal neurons. Specificity controls include pre-treatment of cells with the ErbB receptor inhibitor PD158780 (10 µM, CM+Inh) and pre-incubation of the purified medium with the neutralizing anti-ECD antibody 7215 (CM+Ab). Loading controls include Erk2 and GAPDH protein. As a reference, phosphorylation of Erk2 in response to varying concentration of recombinant NRG2-ECD is shown on the left.

Figure 1h

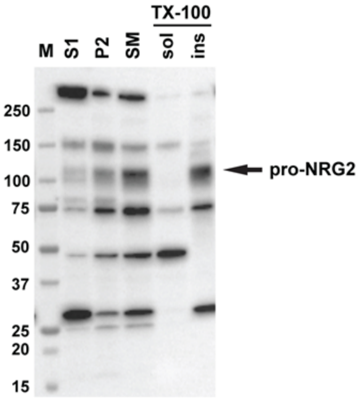

Figure 1i

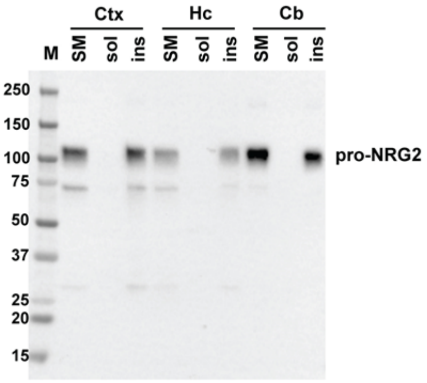

Figure 4a

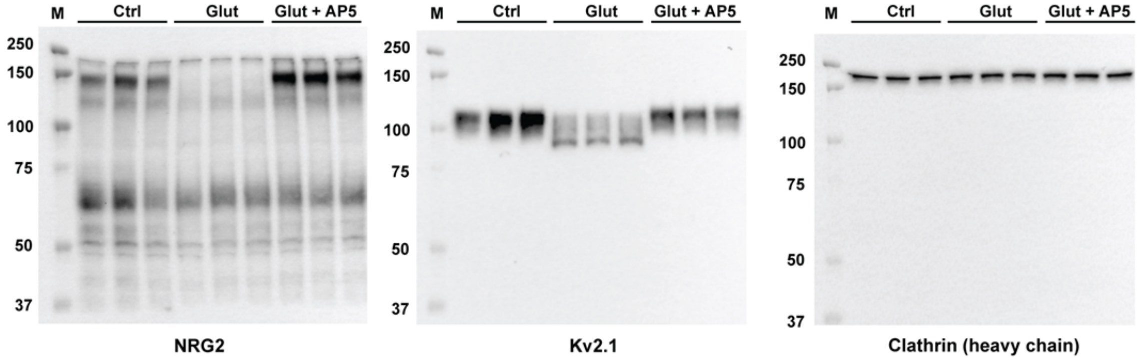

Figure 4c

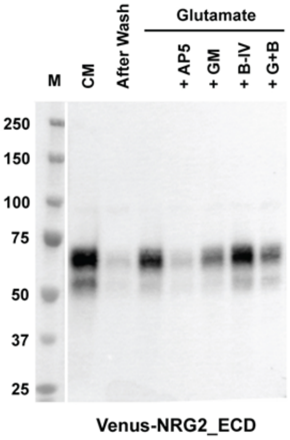

Supplementary Figure 11 Uncropped Western blots (continued on next 2 pages)

Figure 5c

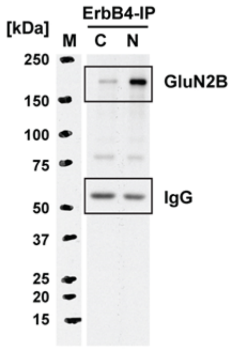

Figure 5d

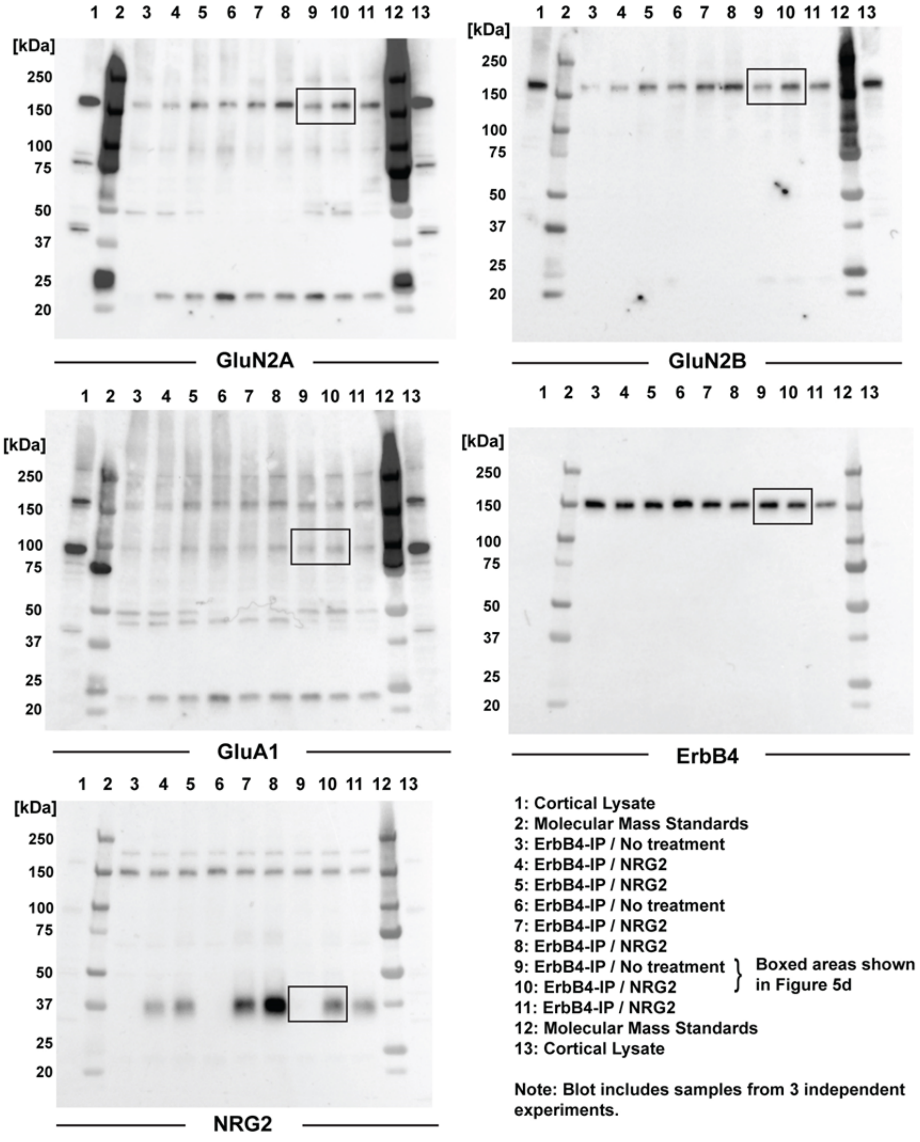

Supplementary Figure 11 Uncropped Western blots (continued)

Supplemental Figure 8

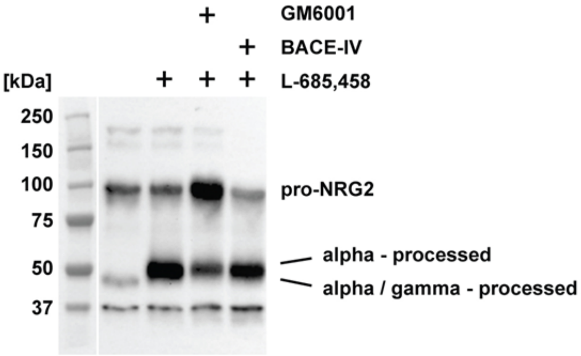

Supplemental Figure 10b

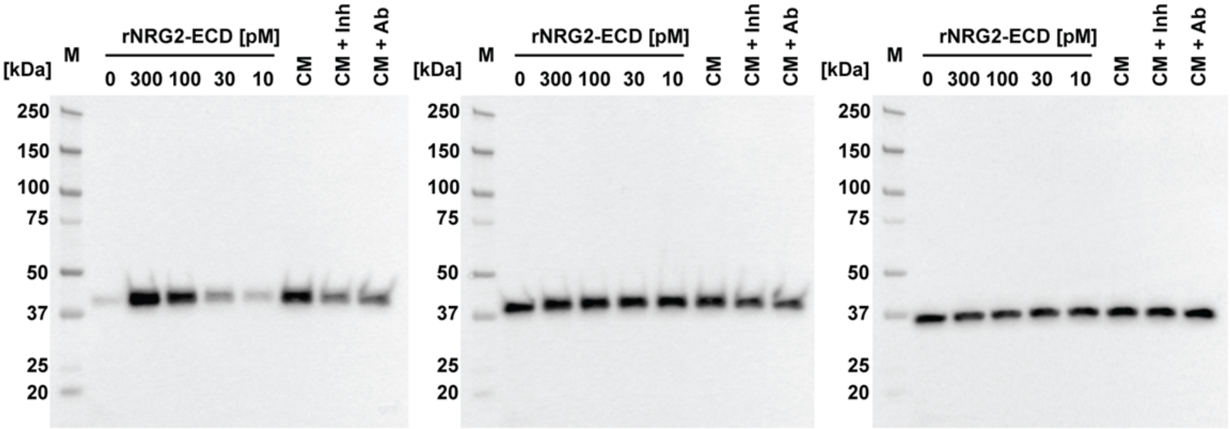

Supplementary Figure 11 Uncropped Western blots (continued)

		Peptides	PepHits	Parsimony Type	Length	Mass	pI	Description
**49 kDa**	KCC2A_RAT	11	17	SUPERSET	478	54650.76	6.61	Calcium/calmodulin-dependent protein kinase type II subunit alpha OS=Rattus norvegicus GN=Camk2a PE=1 SV=1
	KCC2D_RAT	4	6	SUBSET	533	60669.79	6.84	Calcium/calmodulin-dependent protein kinase type II subunit delta OS=Rattus norvegicus GN=Camk2d PE=1 SV=1
	KCC2B_RAT	4	6	SUBSET	542	61104.9	6.73	Calcium/calmodulin-dependent protein kinase type II subunit beta OS=Rattus norvegicus GN=Camk2b PE=1 SV=1
	FLOT1_RAT	3	3	SUPERSET	428	47754.67	6.71	Flotillin-1 OS=Rattus norvegicus GN=Flot1 PE=2 SV=2

		Peptides	PepHits	Parsimony Type	Length	Mass	pI	Description
**150 kDa**	CLH_RAT	4	4	SUPERSET	1675	193187.23	5.5	Clathrin heavy chain 1 OS=Rattus norvegicus GN=Cltc PE=1 SV=3
	**ERBB4_RAT**	**9**	**11**	**DIFFERENTIABLE**	**1308**	**150170.01**	**5.96**	**Receptor tyrosine-protein kinase erbB-4 OS=Rattus norvegicus GN=Erbb4 PE=2 SV=3**
	**NMDE2_RAT**	**3**	**3**	**SUPERSET**	**1482**	**167675.75**	**6.41**	**Glutamate [NMDA] receptor subunit epsilon-2 OS=Rattus norvegicus GN=Grin2b PE=1 SV=1**
	EAA1_RAT	1	1	EQUIVALENT	543	59829.78	8.51	Excitatory amino acid transporter 1 OS=Rattus norvegicus GN=Slc1a3 PE=1 SV=2
	SV2A_RAT	1	1	EQUIVALENT	742	83406.24	5.38	Synaptic vesicle glycoprotein 2A OS=Rattus norvegicus GN=Sv2a PE=1 SV=2
	CNTN1_RAT	2	1	SUPERSET	1021	114278.38	5.77	Contactin-1 OS=Rattus norvegicus GN=Cntn1 PE=1 SV=2
	DCTN1_RAT	1	1	EQUIVALENT	1280	142583.28	5.56	Dynactin subunit 1 OS=Rattus norvegicus GN=Dctn1 PE=2 SV=2
	M3K11_RAT	1	1	SUBSET	850	93621.71	8.71	Mitogen-activated protein kinase kinase kinase 11 OS=Rattus norvegicus GN=Map3k11 PE=2 SV=1
	NCAM1_RAT	1	1	EQUIVALENT	858	95397.51	4.83	Neural cell adhesion molecule 1 OS=Rattus norvegicus GN=Ncam1 PE=1 SV=1

		Peptides	PepHits	Parsimony Type	Length	Mass	pI	Description
**230 kDa**	MYH10_RAT	14	19	SUPERSET	1976	229793.37	5.49	Myosin-10 OS=Rattus norvegicus GN=Myh10 PE=1 SV=1
	MYH11_RAT	2	3	SUBSET	1327	153081.79	5.93	Myosin-11 (Fragments) OS=Rattus norvegicus GN=Myh11 PE=2 SV=3
	MYH9_RAT	6	7	DIFFERENTIABLE	1961	227565.9	5.49	Myosin-9 OS=Rattus norvegicus GN=Myh9 PE=1 SV=3

Supplementary Table 1 Complete list of proteins co-purifying with ErbB4 from metabolizing rat brain synaptosomes following a 10-min treatment with 10 nM NRG2. ErbB4-interacting proteins were co-immunoprecipitated with rabbit monoclonal antibody mAB10 following Triton X-100-solubilization as described previously [3]. Data compiled from the analysis of three different gel slices (see also **Figure 5**).

SUPPLEMENTARY REFERENCES

1. Carraway, K.L., 3rd et al. Neuregulin-2, a new ligand of ErbB3/ErbB4-receptor tyrosine kinases. Nature 387, 512-516 (1997).
2. Rosenbluth, J. Subsurface cisterns and their relationship to the neuronal plasma membrane. J Cell Biol 13, 405-421 (1962).
3. Mitchell, R.M. et al. ErbB4 reduces synaptic GABAA currents independent of its receptor tyrosine kinase activity. Proc Natl Acad Sci U S A 110, 19603-19608 (2013).
